# Supplementary material for: Cortical wiring by synapse type-specific control of local protein synthesis
Source: Science. Author manuscript; Available in PMC 2025 Sep 15. (PMC7618116; doi:10.1126/science.abm7466)
Supplement: Supplementary Material [file EMS208235-suppement-Supplementary_Material.pdf]

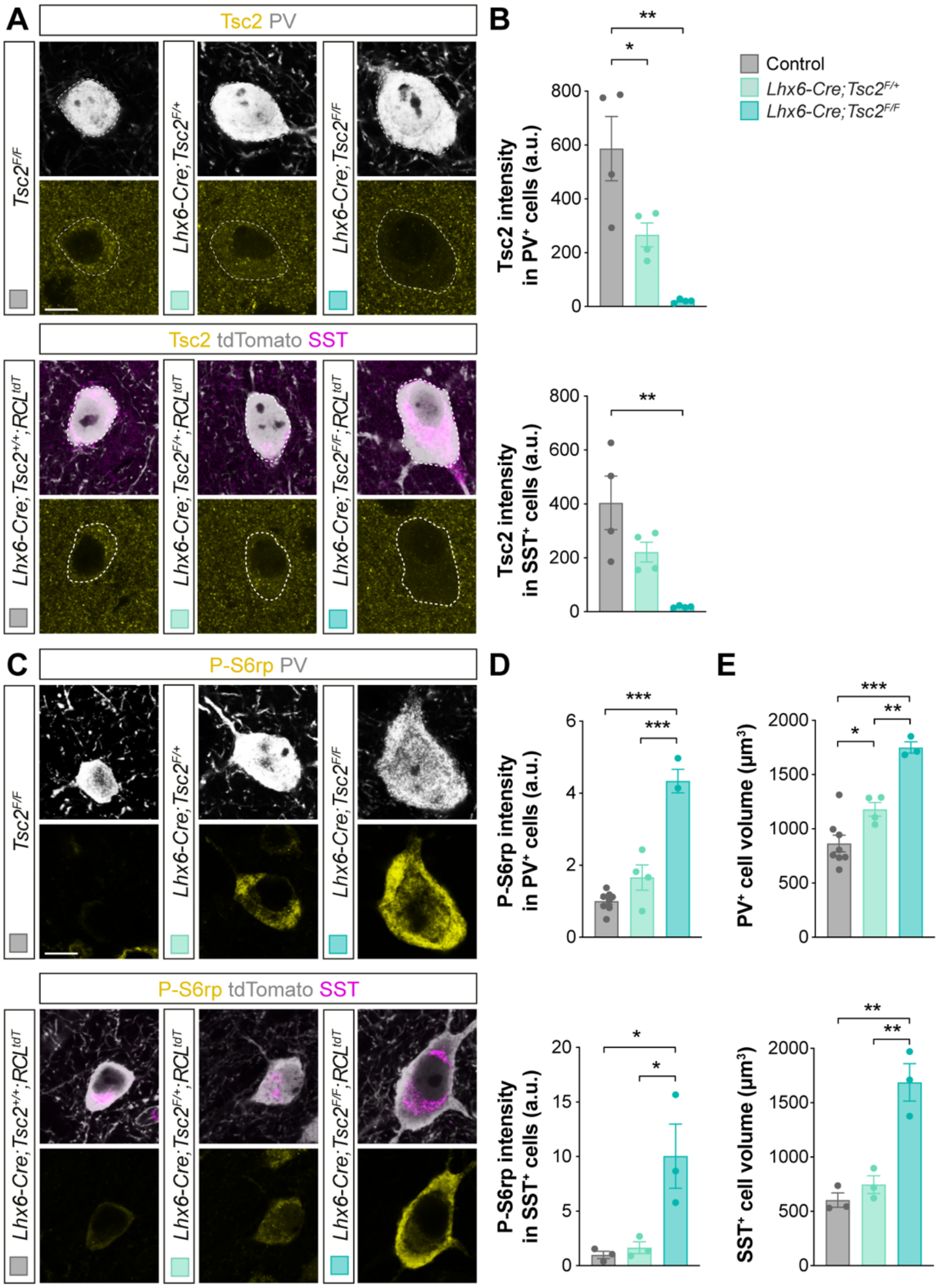

Fig. S1. MGE/POA-derived interneurons exhibit abnormally increased mTOR activation in conditional *Tsc2* mutants. (**A**), Confocal images illustrating the expression of Tsc2 (yellow) in PV+ interneurons (grey) (top) and SST+ (magenta) tdTomato+ (grey) interneurons (bottom) from P18-21 control, heterozygous and homozygous conditional *Tsc2* mutants. (**B**) Quantification of Tsc2 staining intensity in PV+ (top) and SST+ (bottom) interneurons from control, heterozygous and homozygous conditional *Tsc2* mutants. (**C**) Confocal images illustrating phosphorylation of S6rp (P-S6rp, yellow) in PV+ interneurons (grey) (top) and SST+ (magenta) tdTomato+ (grey) interneurons (bottom) from P18-21 control, heterozygous and homozygous conditional *Tsc2*

mutants. (**D**) Quantification of P-S6rp staining intensity in PV^+ (top) and SST^+ (bottom) interneurons from control, heterozygous and homozygous conditional *Tsc2* mutants. (**E**) Quantification of cell volume in PV^+ (top) and SST^+ (bottom) interneurons from control, heterozygous and homozygous conditional *Tsc2* mutants. One-way ANOVA followed by Tukey's multiple comparisons test: *P < 0.05, **P < 0.01, ***P < 0.001 (Tsc2 in PV^+ cells: control *n* = 81 cells from 4 mice; heterozygous, *n* = 80 cells from 4 mice; homozygous, *n* = 71 cells from 4 mice. Tsc2 in SST^+ cells: control *n* = 69 cells from 4 mice; heterozygous, *n* = 70 cells from 4 mice; homozygous, *n* = 81 cells from 4 mice. P-S6rp in PV^+ cells: control *n* = 313 cells from 8 mice; heterozygous, *n* =125 cells from 4 mice; homozygous, *n* = 104 cells from 3 mice. P-S6rp in SST^+ cells: control, *n* = 61 cells from 3 mice; heterozygous, *n* = 75 cells from 3 mice; homozygous, *n* = 61 cells from 3 mice). Data are mean ± s.e.m. Scale bar, 10 μm.

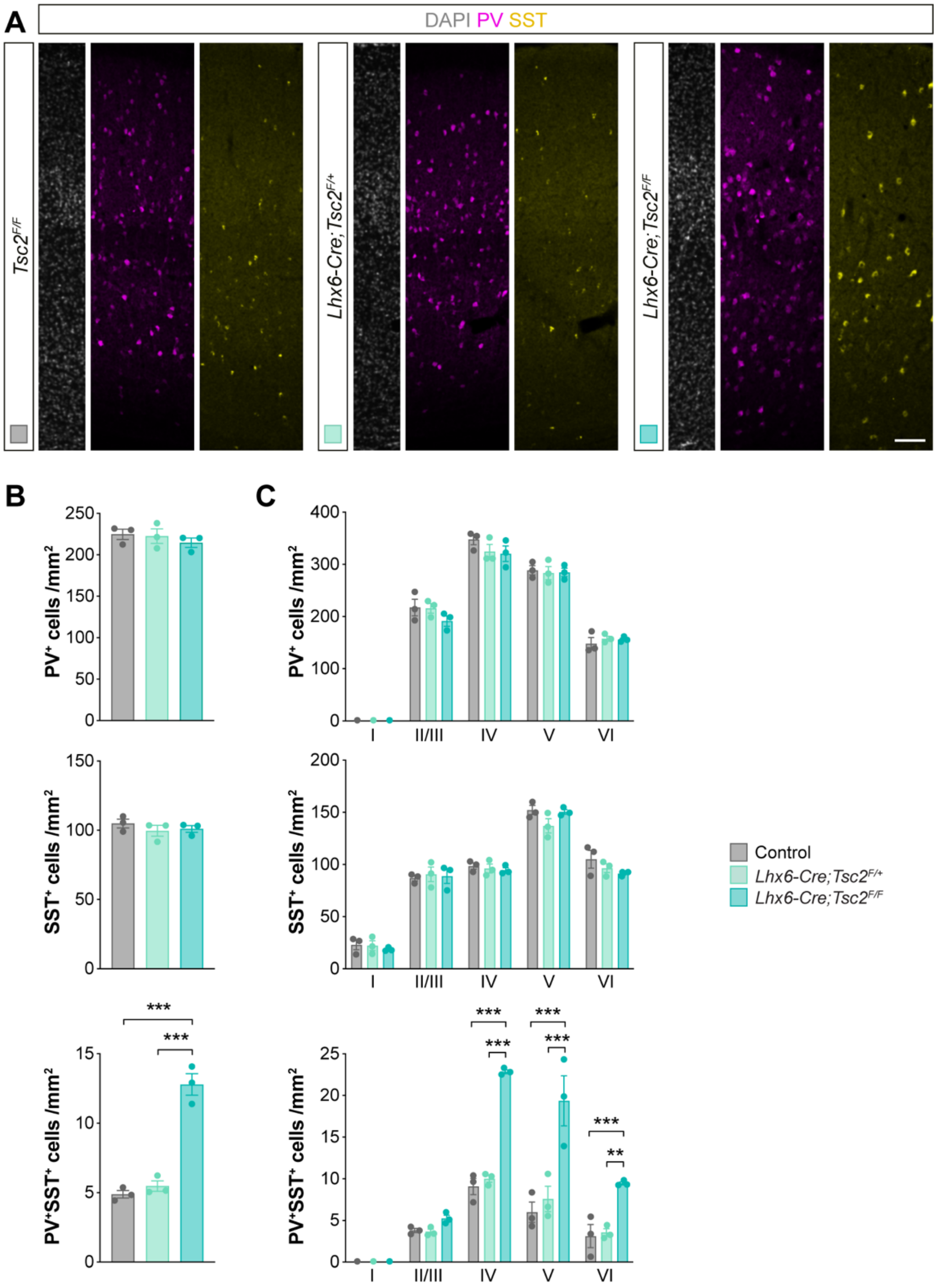

Fig. S2. Density and distribution of MGE/POA-derived interneurons in conditional *Tsc2* mutants. (**A**) Confocal images illustrating the distribution of PV+ (magenta) and SST+ (yellow) interneurons in somatosensory cortex from P18-21 control, heterozygous and homozygous conditional *Tsc2* mutants. (**B-C**) Quantification of the density of PV+ (top), SST+ (middle) and PV+/SST+ (bottom) interneurons in control, heterozygous and homozygous conditional *Tsc2* mutants. One-way ANOVA and Two-way ANOVA followed by Tukey's multiple comparisons test or Kruskal-Wallis followed by Dunn's multiple comparisons test: $^{**}P < 0.01$, $^{***}P < 0.001$

(control $n = 3$ mice; heterozygous, $n = 3$ mice; homozygous, $n = 3$ mice). Data are mean ± s.e.m. Scale bar, 100 μm.

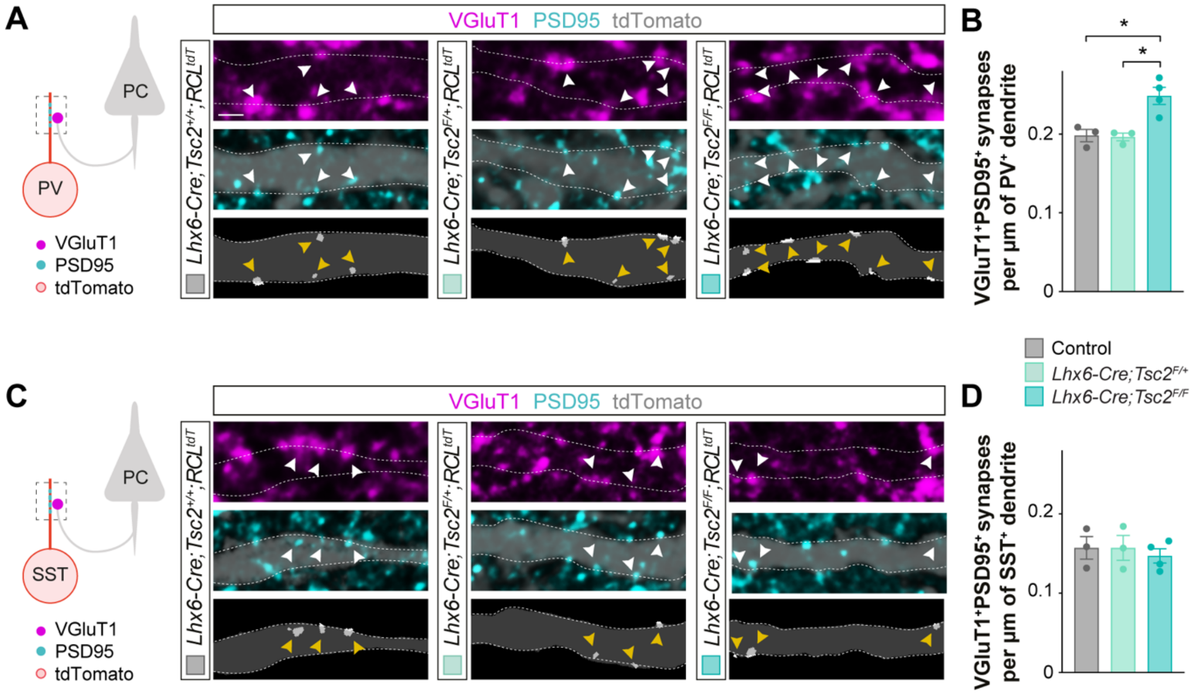

Fig. S3. Conditional deletion of *Tsc2* from PV⁺ and SST⁺ interneurons differentially impact excitatory inputs received by the dendrites of these cells. (**A**) Schematic of synaptic markers analyzed (left). Confocal images (top) and binary images (bottom) illustrating presynaptic VGluT1⁺ puncta (magenta) and postsynaptic PSD95⁺ clusters (cyan) on PV⁺ tdTomato⁺ (grey) dendrites from P18-21 control, heterozygous and homozygous conditional *Tsc2* mutants. (**B**) Quantification of the density of VGluT1⁺PSD95⁺ synapses contacting PV⁺ dendrites (control, *n* = 59 dendrites from 3 mice; heterozygous, *n* = 56 dendrites from 3 mice; homozygous, *n* = 77 dendrites from 4 mice). (**C**) Schematic of synaptic markers analyzed (left). Confocal images (top) and binary images (bottom) illustrating presynaptic VGluT1⁺ puncta (magenta) and postsynaptic PSD95⁺ clusters (cyan) on SST⁺ tdTomato⁺ (grey) dendrites from P18-21 control, heterozygous and homozygous conditional *Tsc2* mutants. (**D**) Quantification (right) of the density of VGluT1⁺PSD95⁺ synapses contacting SST⁺ dendrites (control, *n* = 55 dendrites from 3 mice; heterozygous, *n* = 61 dendrites from 3 mice; homozygous, *n* = 71 cells from 4 mice). One-way ANOVA followed by Tukey's multiple comparisons test: *$P < 0.05$. Data are mean ± s.e.m. Scale bar, 1 μm.

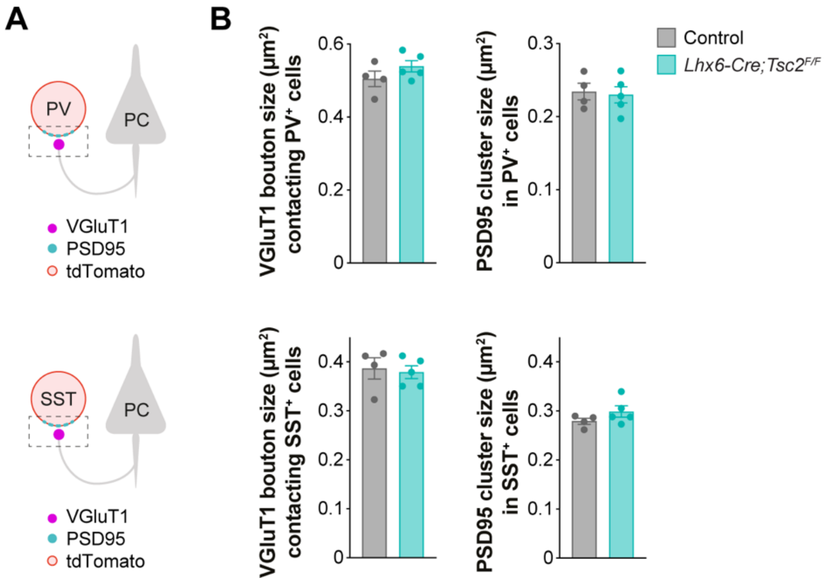

Fig. S4. Conditional deletion of *Tsc2* from PV⁺ and SST⁺ interneurons does not seem to impact synaptic size. (**A**) Schematic of synaptic markers analyzed (left). Quantification of the size of VGluT1⁺ boutons (middle) and PSD95⁺ clusters (right) on PV⁺ cells from P18-21 control and homozygous conditional *Tsc2* mutants (control, *n* = 106 cells from 4 mice; homozygous, *n* = 131 cells from 5 mice). (**B**) Schematic of synaptic markers analyzed (left). Quantification of the size of VGluT1⁺ boutons (middle) and PSD95⁺ clusters (right) on SST⁺ cells from P18-21 control and homozygous conditional *Tsc2* mutants (control, *n* = 57 cells from 4 mice; homozygous, *n* = 56 cells from 5 mice). Two-tailed Student's unpaired t-test. Data are mean ± s.e.m.

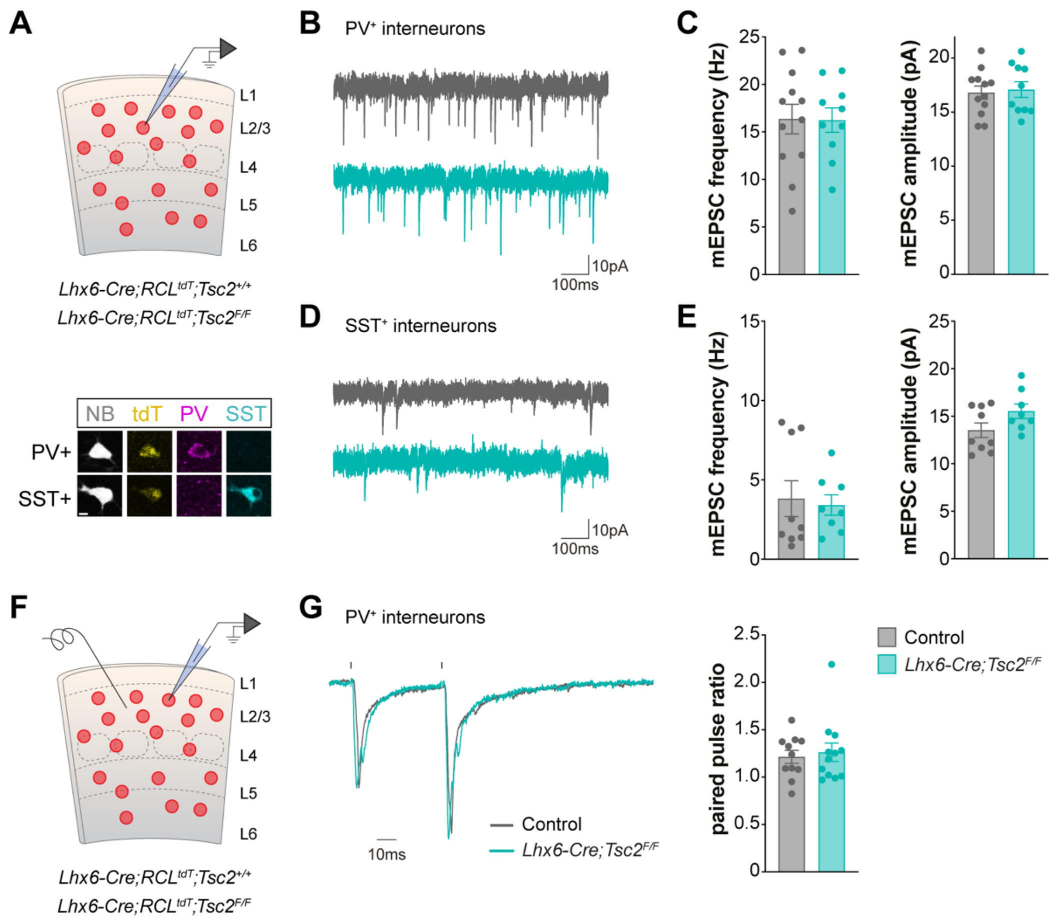

Fig. S5. Conditional deletion of *Tsc2* from PV⁺ and SST⁺ interneurons does not increase the functional excitatory synapses received by these cells. (**A**) Schematic of experimental design (top) and post-recording labelling of neurobiotin (NB, grey) -filled tdTomato⁺ (yellow) cells with PV (magenta) and SST (cyan) (bottom). (**B**) Example traces of mEPSCs recorded from PV⁺ interneurons from P18-21 control and homozygous conditional *Tsc2* mutants. (**C**) Quantification of the frequency (left) and amplitude (right) of mEPSCs from PV⁺ interneurons (control, *n* = 12 cells from 7 mice; homozygous, *n* = 10 cells from 4 mice). (**D**) Example traces of mEPSCs recorded from SST⁺ interneurons from P18-21 control and homozygous conditional *Tsc2* mutants. (**E**) Quantification of the frequency (left) and amplitude (right) of mEPSCs from SST⁺ interneurons (control, *n* = 9 cells from 6 mice; homozygous, *n* = 8 cells from 4 mice). (**F**) Schematic of experimental design. (**G**) Example traces with stimulation indicated above the trace (left) and quantification (right) of the paired-pulse ratio of eEPSCs from PV⁺ interneurons in control and homozygous conditional *Tsc2* mutants (control, *n* = 11 cells from 7 mice; homozygous, *n* = 12 cells from 6 mice). Two-tailed Student's unpaired t-test or Mann-Whitney test. Data are mean ± s.e.m.

A

	Lhx6-Cre;Tsc2$^{+/+}$	*Lhx6-Cre;Tsc2*$^{F/F}$	
**Fast-spiking interneurons**			
RMP (mV)	-62.61 ± 2.15	-63.21 ± 0.97	
TC (ms)	11.73 ± 0.79	11.00 ± 0.74	
IR (MΩ)	104.67 ± 10.23	71.26 ± 5.55	*
Ih sag (mV)	-4.49 ± 0.54	-7.42 ± 0.60	**
AP-threshold (mV)	-34.94 ± 1.24	-36.07 ± 0.59	
APmax (mV)	26.72 ± 1.31	31.57 ± 1.29	*
Half-width (ms)	0.31 ± 0.01	0.36 ± 0.01	**
Rise time 20-80 (ms)	0.09 ± 0.00	0.10 ± 0.00	**
Decay time 80-20 (ms)	0.12 ± 0.00	0.15 ± 0.01	**
Delay to AP (ms)	194.65 ± 33.98	184.72 ± 32.59	
MFF (Hz)	167.27 ± 9.36	171.18 ± 11.70	
fAHP amplitude (mV)	23.45 ± 1.12	23.05 ± 0.67	
Capacitance (pF)	119.21 ± 7.71	158.14 ± 10.57	**
**Non-fast-spiking interneurons**			
RMP (mV)	-57.43 ± 2.15	-56.33 ± 1.92	
TC (ms)	27.96 ± 2.95	42.57 ± 4.31	**
IR (MΩ)	242.10 ± 29.56	189.01 ± 19.15	
Ih sag (mV)	-7.73 ± 0.98	-11.40 ± 0.93	*
AP-threshold (mV)	-39.00 ± 2.29	-35.69 ± 1.19	
APmax (mV)	32.31 ± 2.08	38.88 ± 1.51	*
Half-width (ms)	0.48 ± 0.02	0.56 ± 0.02	*
Rise time 20-80 (ms)	0.12 ± 0.00	0.14 ± 0.00	*
Decay time 80-20 (ms)	0.21 ± 0.02	0.26 ± 0.01	*
Delay to AP (ms)	144.12 ± 26.39	311.51 ± 27.15	***
MFF (Hz)	61.67 ± 5.27	68.00 ± 5.47	
fAHP amplitude (mV)	17.42 ± 2.07	18.61 ± 0.59	
Capacitance (pF)	125.84 ± 13.74	231.37 ± 19.81	***

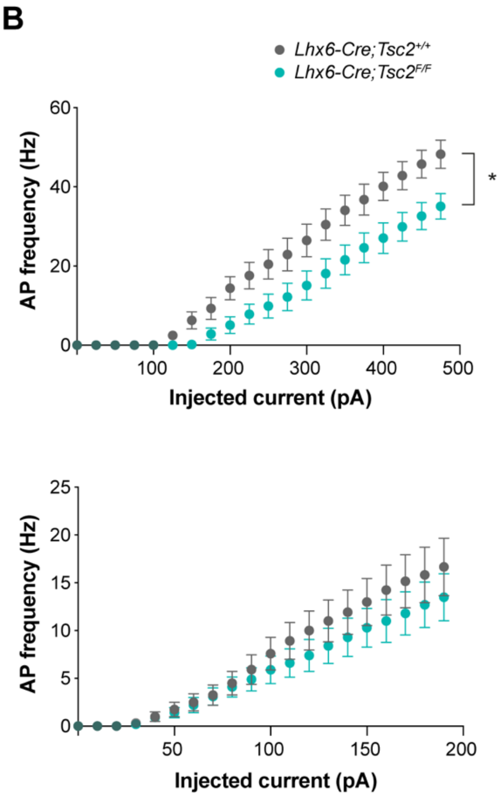

Fig. S6. Intrinsic properties of MGE/POA-derived interneurons in conditional *Tsc2* mutants. (**A**) Intrinsic electrophysiological properties of fast-spiking (top, putative PV$^+$ interneurons) and non-fast-spiking (bottom, putative SST$^+$ interneurons) interneurons from P18-21 control and homozygous conditional *Tsc2* mutants. RMP: resting membrane potential, TC: time constant, IR: input resistance, AP: action potential, MFF: maximum firing frequency, fAHP: fast afterhyperpolarization. (**B**) Excitability curves showing the spike frequency of fast-spiking (top) and non-fast-spiking (bottom) interneurons from P21 control and homozygous conditional *Tsc2* mutants in response to current injections. Two-way ANOVA with repeated measures: *$P < 0.05$ (fast-spiking interneurons: control, n = 15 cells from 8 mice; homozygous, n = 11 cells from 4 mice. Non-fast-spiking interneurons: control, n = 12 cells from 6 mice; homozygous, n = 10 cells from 4 mice). Data are mean ± s.e.m.

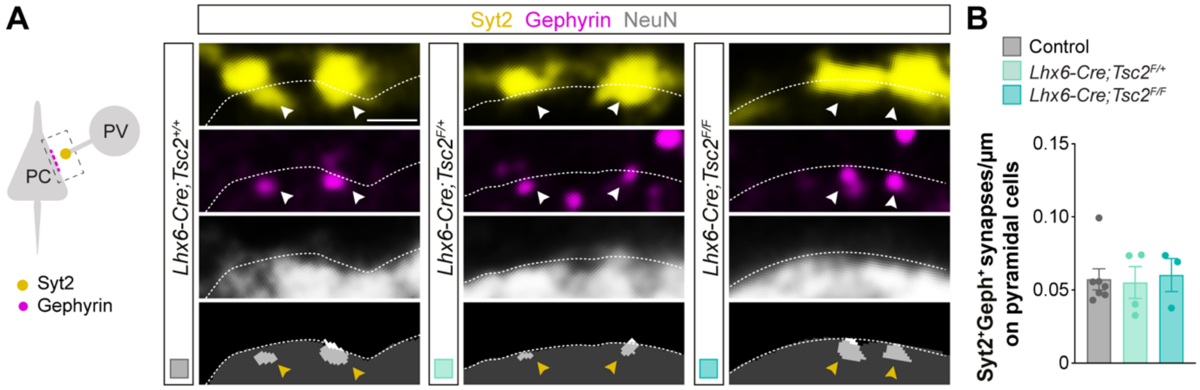

Fig. S7. *Tsc2* deletion does not affect the synaptic output of PV⁺ basket interneurons. (**A**) Schematic of synaptic markers analyzed (left). Confocal images (top) and binary images (bottom) illustrating presynaptic Syt2⁺ puncta (yellow) and postsynaptic Gephyrin⁺ clusters (magenta) in NeuN⁺ pyramidal cells (grey) from P18-21 control, heterozygous and homozygous conditional *Tsc2* mutants (right). (**B**) Quantification of the density of Syt2⁺Gephyrin⁺ synapses contacting pyramidal cells (control, n = 112 cells from 7 mice; heterozygous, n = 71 cells from 4 mice; homozygous, n = 36 cells from 3 mice). Kruskal-Wallis followed by Dunn's multiple comparisons test. Data are mean ± s.e.m. Scale bar, 1 μm.

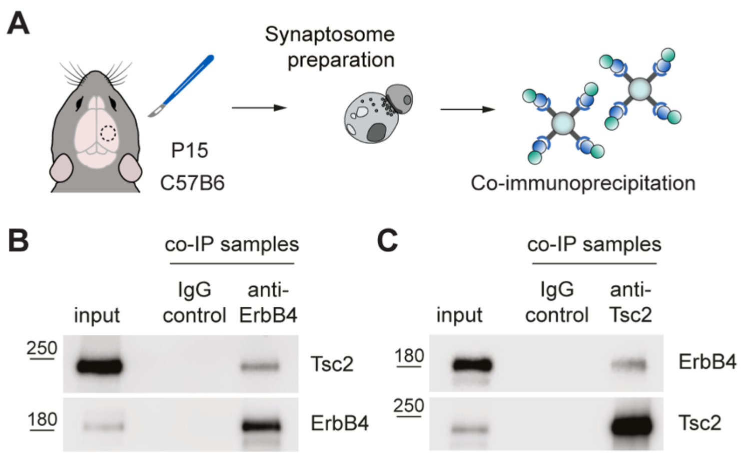

Fig. S8. ErbB4 and Tsc2 associate at the synapse at the time of synaptogenesis. (**A**) Schematic of experimental design. (**B**) Western blot for Tsc2 and ErbB4 of anti-ErbB4 co-immunoprecipitation samples from cortical synaptosomes from P15 C57B6 mice (*n* = 3 independent co-immunoprecipitation experiments). (**C**) Western blot for Tsc2 and ErbB4 of anti-Tsc2 co-immunoprecipitation samples from cortical synaptosomes from P15 C57B6 mice (*n* = 3 independent co-immunoprecipitation experiments).

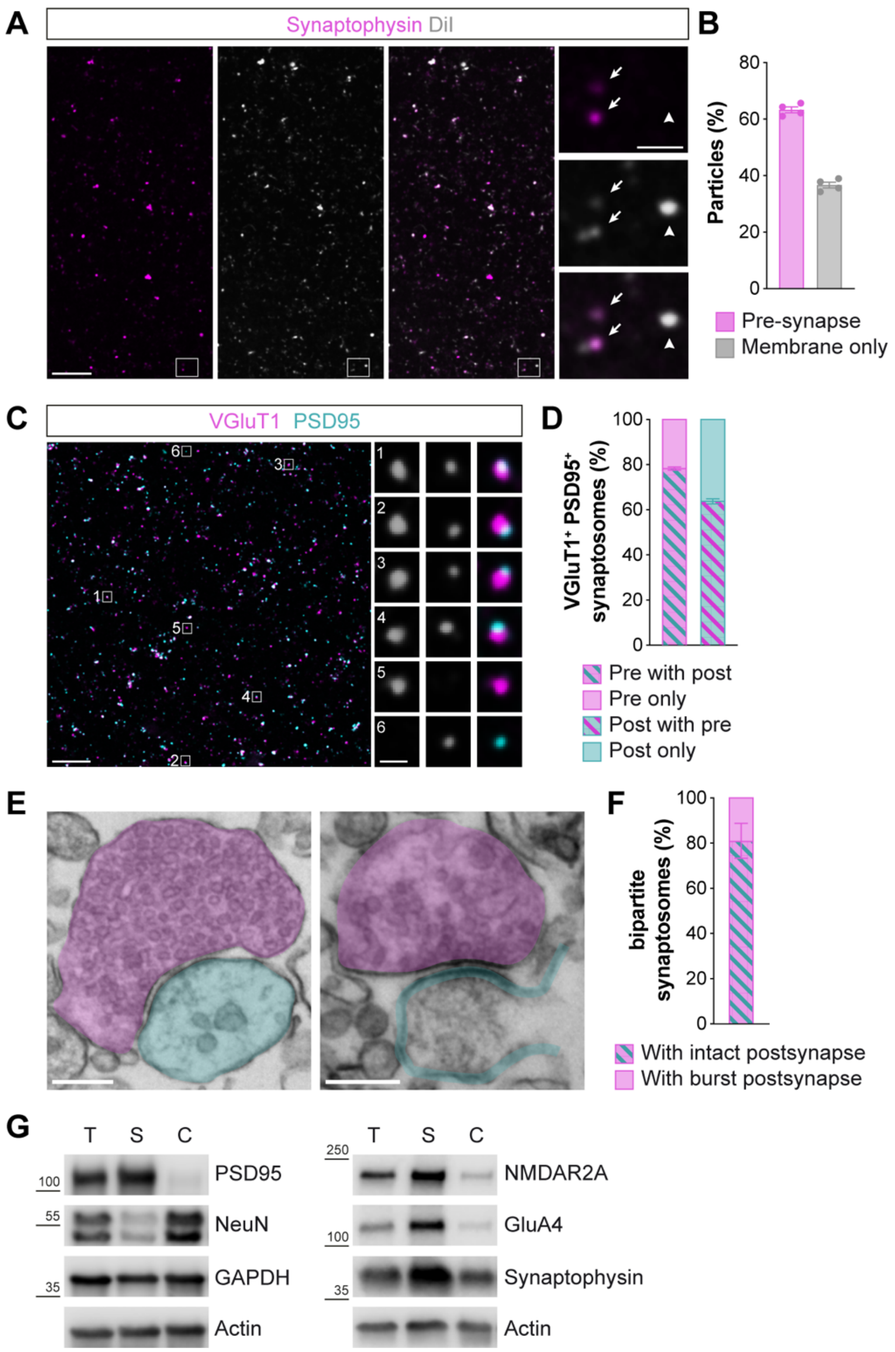

Fig. S9. Evaluation of the quality of synaptosome preparations. (**A**) Confocal images illustrating synaptosome preparations from P21 C57B6 mice stained with DiI (grey) and immunohistochemistry against Synaptophysin+ (magenta). In insets (right), arrows indicate Synaptophysin+DiI+ pre-synaptic particles, and arrowheads indicate Synaptophysin−DiI+ membrane contaminants, including residual postsynapses. (**B**) Percentage of particles corresponding to pre-synapse or membrane contaminant. Pre-synaptic particles include Synaptophysin+DiI− synaptosomes (*n* = 20 fields of view from 4 mice). (**C**) Confocal images illustrating VGluT1+ (magenta) PSD95+ (cyan) synaptosomes from P21 C57B6 mice. Insets (right)

show single-channel images for VGluT1 (left, grey), PSD95 (middle, grey) and merge images (right) of examples of bipartite (1-4), pre-only (5) and post-only (6) synaptosomes. (**D**) Percentages of VGluT1^{+}PSD95^{+} synaptosomes that are bipartite, pre-only or post-only (n = 23,132 synaptosomes from 4 mice). (**E**) Transmission Electron Microscopy (TEM) images illustrating synaptosomes from P21 C57B6 mice. Presynapses are highlighted in magenta and postsynapses in cyan, with examples of intact (left) and burst (right) postsynapses. (**F**) Percentages of bipartite synaptosomes with intact membrane-enclosed or burst postsynaptic side (n = 416 bipartite synaptosomes from 2 electron micrographs from 4 mice). (**G**) Western blot for presynaptic (synaptophysin), postsynaptic (PSD95, NMDAR2A, GluA4), cytosolic (GAPDH, NeuN) and ubiquitous (actin) proteins in total homogenates (T), synaptic (S) and cytosolic (C) fractions from P21 C57B6 mice. Data are mean ± s.e.m. Scale bar, (A) 10 μm and 2 μm (insets), (C) 10 μm and 1 μm (insets), (E) 200 nm.

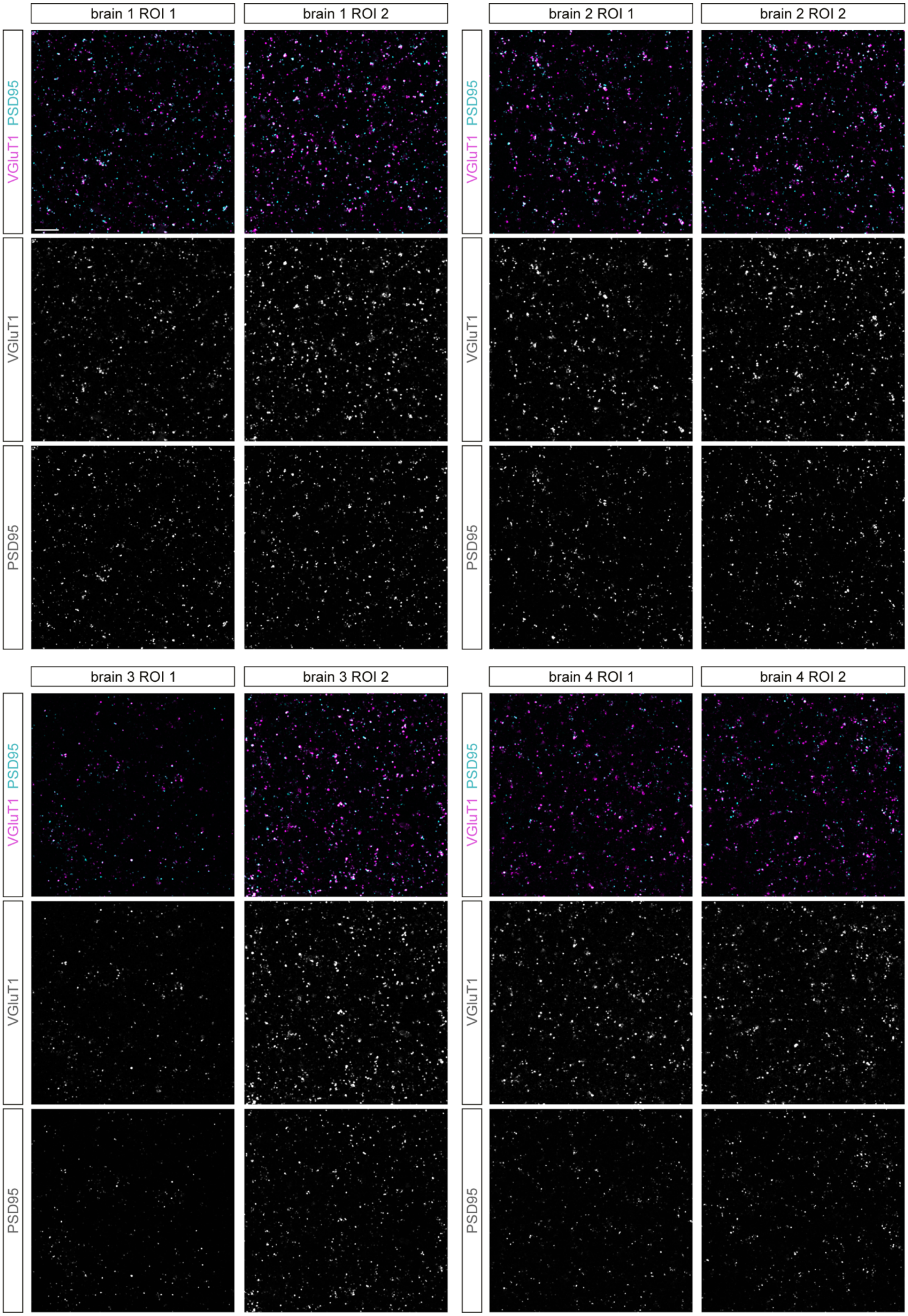

brain 1 ROI 1
brain 1 ROI 2
brain 2 ROI 1
brain 2 ROI 2
VGluT1 PSD95
VGluT1
PSD95
brain 3 ROI 1
brain 3 ROI 2
brain 4 ROI 1
brain 4 ROI 2
VGluT1 PSD95
VGluT1
PSD95

Fig. S10. Examples of confocal images used to quantify VGluT1 and PSD95 immunofluorescence in synaptosomes. P21 synaptosomes from C57B6 mice were plated, stained, and imaged using a confocal microscope. Individual black and white images are provided for each channel, and a merged image with VGluT1 in magenta and PSD95 in cyan. Scale bar, 10 μm.

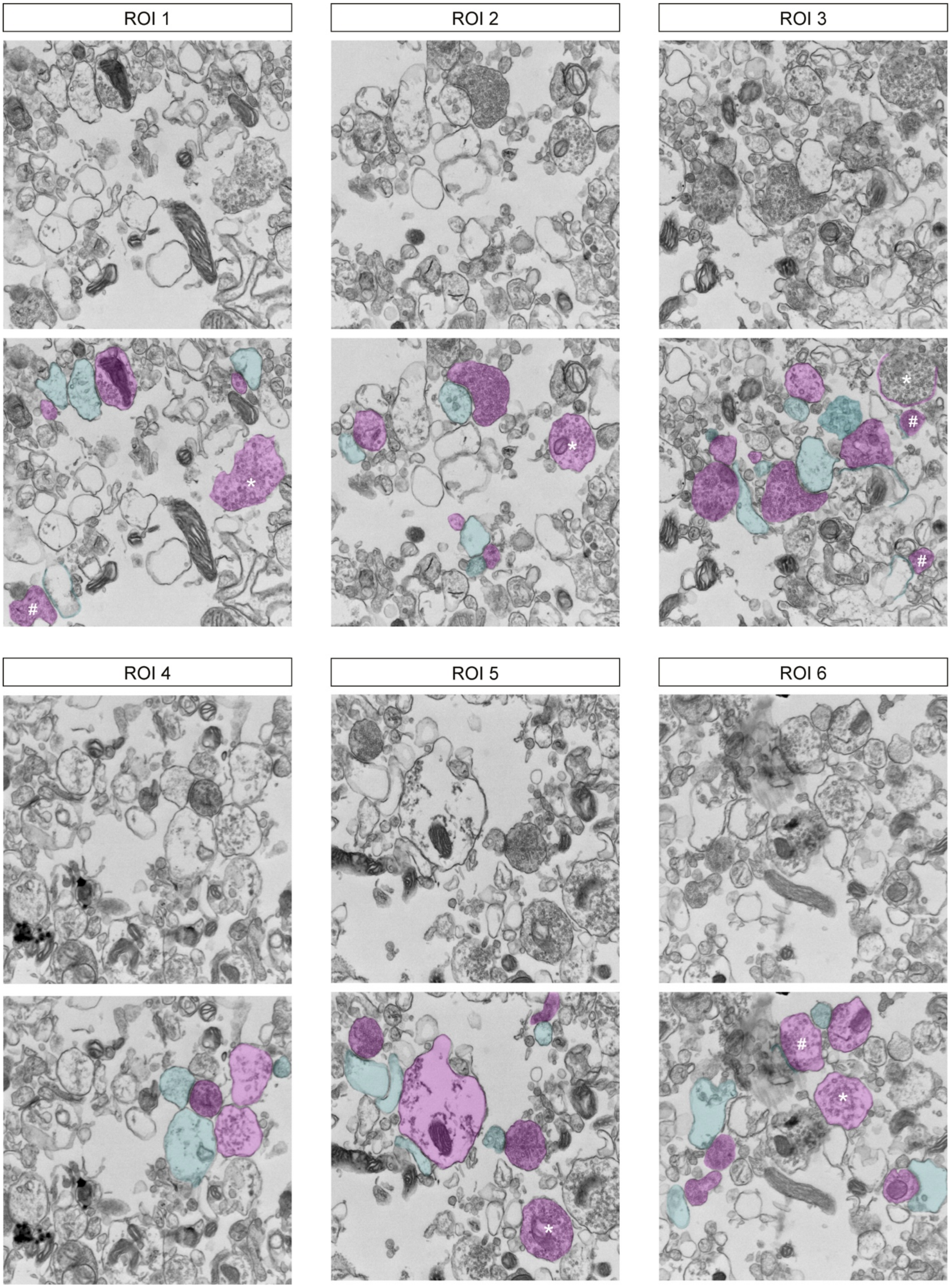

ROI 1
ROI 2
ROI 3
ROI 4
ROI 5
ROI 6

Fig. S11. Examples of TEM micrographs used to quantify bipartite synaptosomes. Examples of TEM images used to quantify intact and burst postsynapses in synaptosomes obtained from P21 C57B6 mice. Synaptosomes were identified by the presence of a presynaptic terminal with synaptic vesicles. The coloured images highlight presynapses in magenta and postsynapses in cyan. (*) indicates presynapses without an obvious postsynapse on the plane analysed; (#) indicates presynapses with a burst postsynapse. Scale bar, 500 nm.

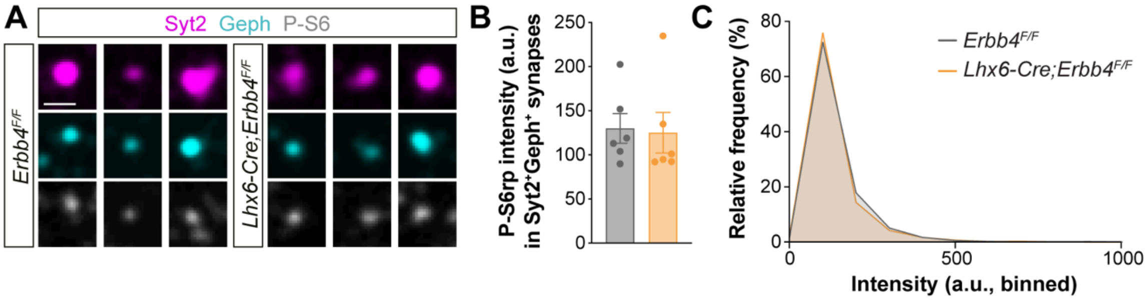

Fig. S12. *Erbb4* deletion does not affect the phosphorylation of S6rp in PV⁺ output synapses. (**A**) Confocal images illustrating phosphorylation of S6rp (P-S6rp, grey) in Syt2$^+$ (magenta) Geph$^+$ (cyan) synaptosomes from P21 homozygous *Erbb4* mice and their control littermates. (**B**) Quantification of P-S6rp staining intensity in Syt2$^+$Geph$^+$ synaptosomes. (**C**) Relative frequency distribution of P-S6rp staining intensity in Syt2$^+$Geph$^+$ synaptosomes (control, n = 5,327 synaptosomes from 6 mice, homozygous, n = 5,072 synaptosomes from 6 mice). Mann-Whitney test. Data are mean ± s.e.m. Scale bar, 1 μm.

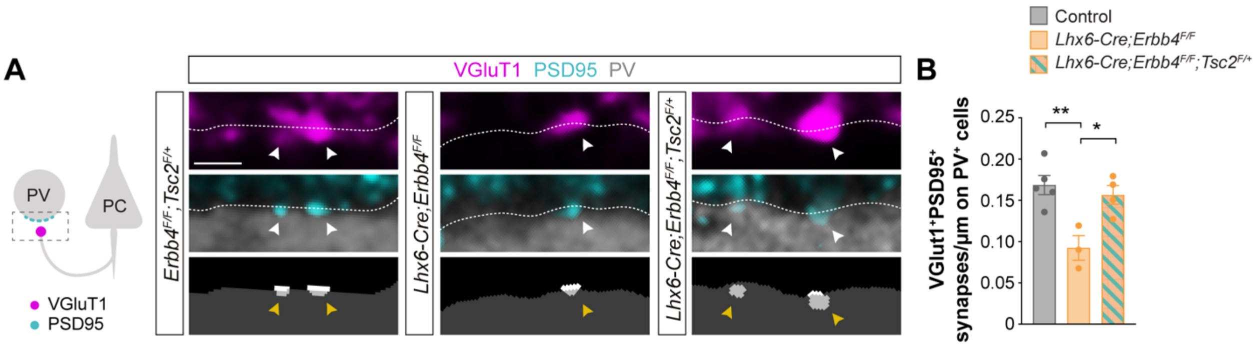

Fig. S13. *Tsc2* deletion rescues synaptic loss in *Erbb4* conditional mutants. (**A**) Schematic of synaptic markers analyzed (left). Confocal images (top) and binary images (bottom) illustrating presynaptic VGluT1$^+$ puncta (magenta) and postsynaptic PSD95$^+$ clusters (cyan) in PV$^+$ interneurons (grey) from P21 *Lhx6-Cre;Erbb4$^{F/F}$;Tsc2$^{F/+}$* mice, *Lhx6-Cre;Erbb4$^{F/F}$* and their control littermates. (**B**) Quantification of the density of VGluT1$^+$PSD95$^+$ synapses contacting PV$^+$ interneurons (control, n = 124 cells from 5 mice; *Lhx6-Cre;Erbb4$^{F/F}$*, n = 67 cells from 3 mice; *Lhx6-Cre;Erbb4$^{F/F}$;Tsc2$^{F/+}$*, n = 85 cells from 4 mice). ANOVA followed by Tukey's multiple comparisons test: *P < 0.05, **P < 0.01. Data are mean ± s.e.m. Scale bar, 1 μm.

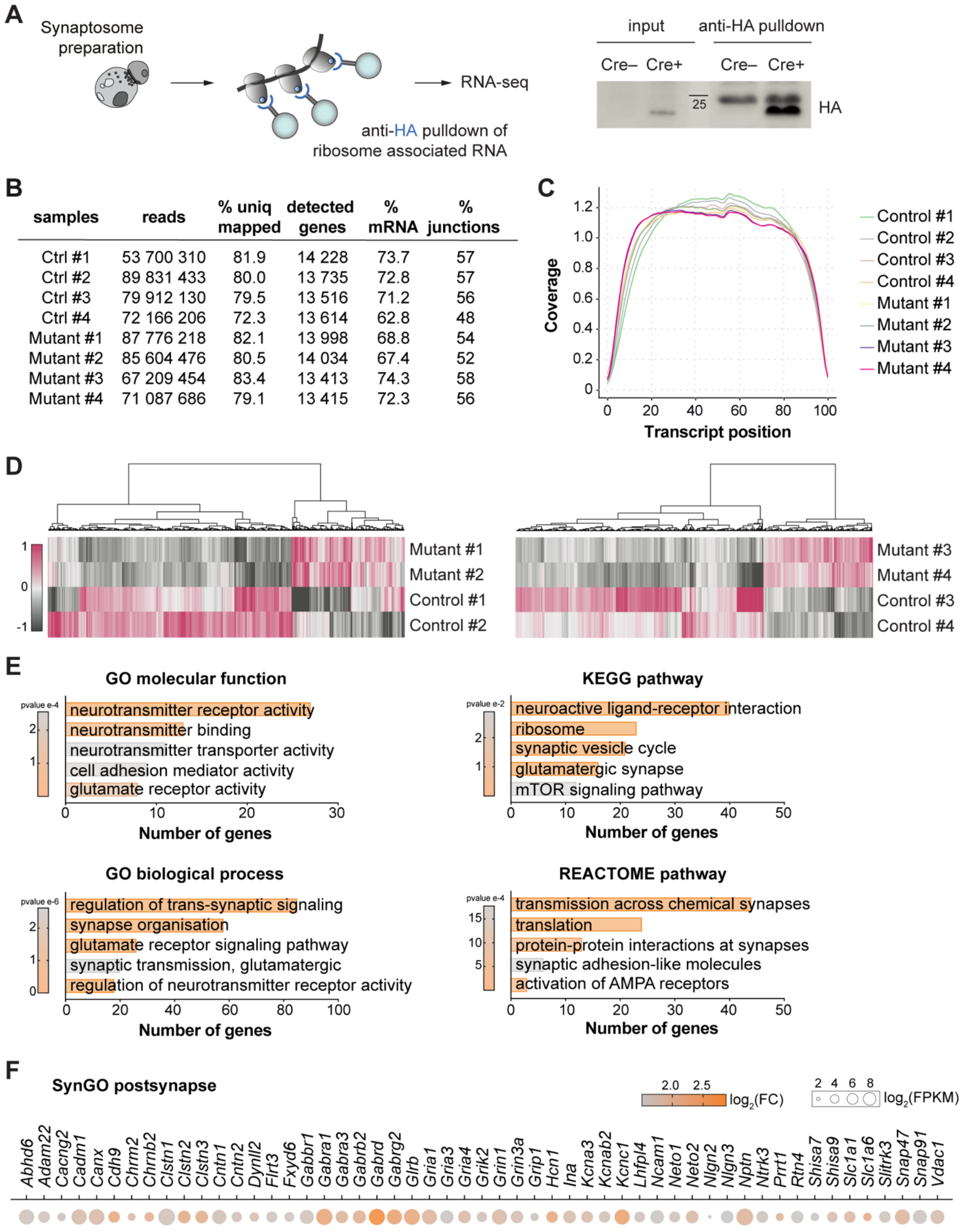

Fig. S14. RNA sequencing data and gene ontology analysis of downregulated genes in *Erbb4* conditional mutants. (**A**) Schematic of experimental design (left). Western blot for HA of anti-HA pulldown samples from cortical synaptosomes from P15 *Lhx6-Cre;Rpl22*$^{HA/HA}$ mice (*n* = 2

independent co-immunoprecipitation experiments) (right). Note that the top band present in both Cre– and Cre+ anti-HA pulldown samples corresponds to IgGs from the pulldown antibodies. (**B**) FastQC analysis of RNA sequencing data for each replicate, showing the total number of reads, % of reads uniquely mapped to the reference genome, number of detected genes, % of mRNA representation and % of reads mapped to exon-exon junctions. (**C**) 5' to 3' coverage plot showing the percentage of read bases across the transcript length. (**D**) Heatmaps showing significantly differentially expressed genes from two RNA sequencing batches from P15 *Lhx6-Cre;Erbb4*$^{F/F}$*;Rpl22*$^{HA/HA}$ (conditional *Erbb4* mutants) cortical synaptosomes compared to *Lhx6-Cre;Erbb4*$^{+/+}$*;Rpl22*$^{HA/HA}$ controls. Heatmap values are Deseq2 normalized counts. (**E**) Selected Gene Ontology (GO) terms, REACTOME and KEGG pathways significantly enriched in the dataset of downregulated genes in *Erbb4* mutants compared to controls. (**F**) Heatmap illustrating both control FPKM values and fold-change in conditional *Erbb4* mutants for genes from "postsynaptic specialization" and "postsynaptic membrane" SynGO categories.

A

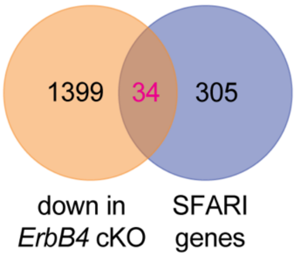

B

Gene symbol	fold-down *ErbB4*$^{F/F}$	Gene score
Atp1a3	2.8	S
Slc6a1	2.64	1
Cntnap4	2.45	2
Arx	2.25	1
Hcn1	2.24	S
Cntnap2	2.05	S
Oxtr	2.03	2
Cntnap3	1.99	2
Reln	1.96	1
Dhcr7	1.95	1
Slc9a6	1.94	1
Gria1	1.94	2
Scn1a	1.93	1
Slc12a5	1.89	2
Nrxn3	1.88	1
Grin1	1.83	1
Eef1a2	1.81	S
Dpp10	1.73	2
Ywhag	1.68	S
Cnr1	1.67	2
Grik2	1.66	2
Ntng1	1.66	S
Mboat7	1.62	1
Grip1	1.61	2
Kcna2	1.6	S
Cntn6	1.58	2
Gabrg3	1.58	2
Atp1a1	1.57	S
Nlgn2	1.57	1
Gria3	1.57	S
Kirrel3	1.57	2
Cacna2d3	1.53	1
Syn1	1.52	1
Nlgn3	1.51	1

Fig. S15. Autism-associated genes from the SFARI Gene database are enriched among genes downregulated in *Erbb4* conditional mutants. (**A**) Venn diagram illustrating the overlap between genes downregulated in *Erbb4* conditional mutants and highly-reported genes from the SFARI Gene database (1.58-fold enrichment). Fisher's exact test P=1.51 e^{-2}. (**B**) List of highly-reported SFARI genes downregulated in *ErbB4* conditional mutants (SFARI Gene scoring: S, syndromic; 1, high confidence; 2, strong candidate).

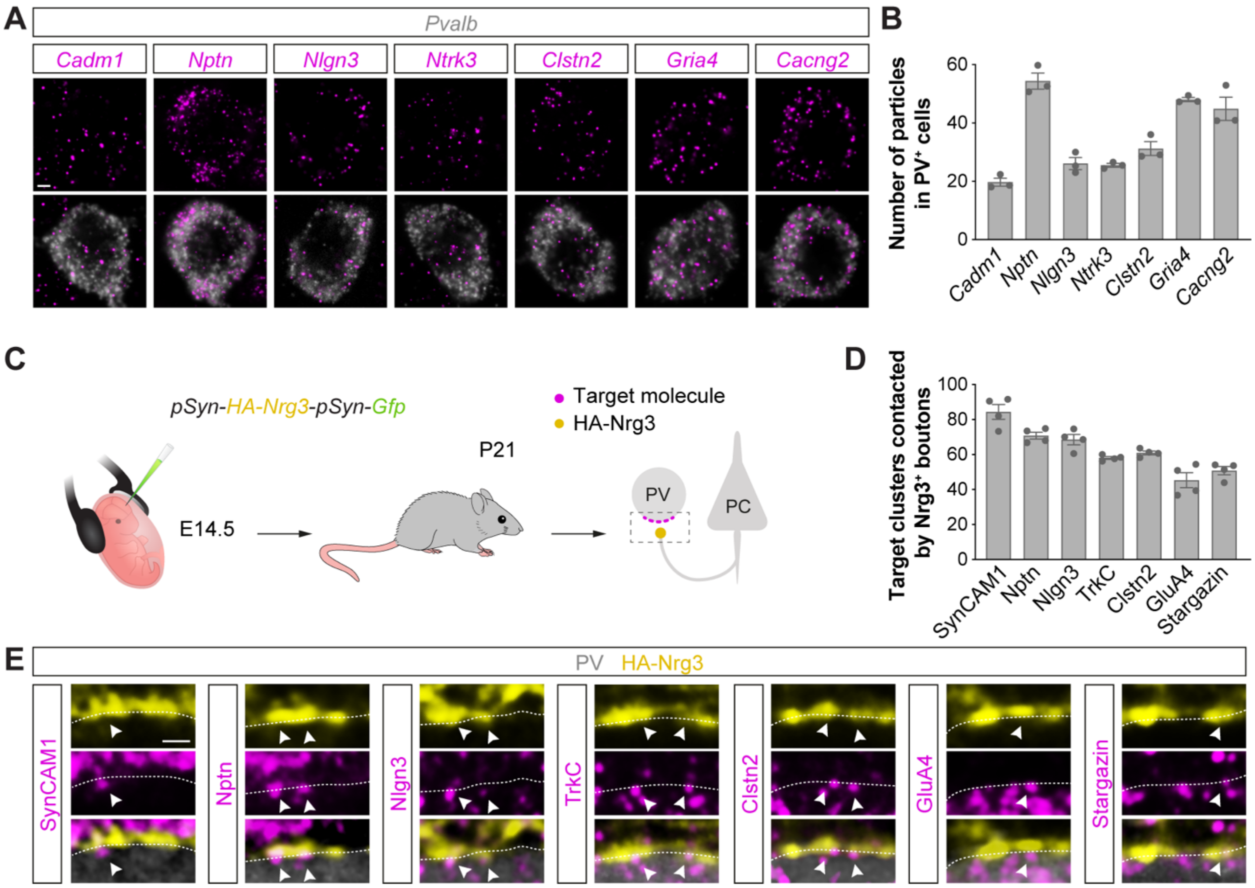

Fig. S16. ErbB4 targets are expressed in developing cortical PV+ interneurons and are apposed to Nrg3+ excitatory synapses innervating PV+ cells. (**A**) Confocal images of target mRNAs (magenta) and *Pvalb* mRNA (grey) from single-molecule fluorescent in situ hybridization in P15 C57B6 mice. (**B**) Number of mRNA particles per PV+ cell soma. *Cadm1*, *n* = 60 cells from 3 mice; *Nptn*, *n* = 66 cells from 3 mice; *Nlgn3*, *n* = 60 cells from 3 mice; *Ntrk3*, *n* = 60 cells from 3 mice; *Clstn2*, *n* = 60 cells from 3 mice; *Gria4*, *n* = 71 cells from 3 mice; *Cacng2*, *n* = 66 cells from 3 mice. (**C**) Schematic of experimental design. (**D**) Proportion of target protein clusters contacted by Nrg3+ presynaptic boutons in PV+ interneurons. SynCAM1, *n* = 26 cells from 4 mice; Nptn, *n* = 26 cells from 4 mice; Nlgn3, *n* = 26 cells from 4 mice; TrkC, *n* = 26 cells from 4 mice; Clstn2, *n* = 26 cells from 4 mice; GluA4, *n* = 26 cells from 4 mice; Stargazin, *n* = 24 cells from 4 mice. (**E**) Confocal images illustrating protein clusters (magenta) for the 7 targets at the surface of PV+ cell somas (grey), in close apposition to HA-Nrg3+ presynaptic boutons (yellow) from pyramidal cells electroporated with the plasmid *pSyn-HA-Nrg3-pSyn-Gfp* at embryonic day E14.5. Data are mean ± s.e.m. Scale bar, 3 μm (A) and 1 μm (E).

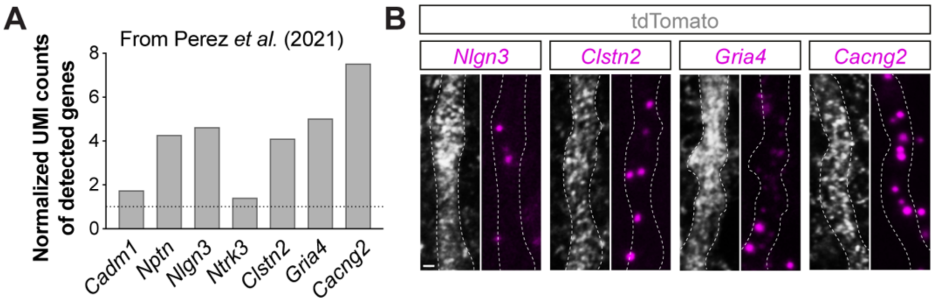

Fig. S17. ErbB4 targets are present in dendrites of developing PV⁺ interneurons. (**A**) Normalized Unique Molecular Identifier (UMI) counts of target genes in dendrites of GABAergic neurons from DIV14 rat primary cultures from Supplementary File 3 of Ref. 24. (**B**) Confocal images of target mRNAs (magenta) in PV^+ tdT^+ (grey) dendrites from P15 *Lhx6-Cre;tdT* mice. Scale bar, 1 μm.

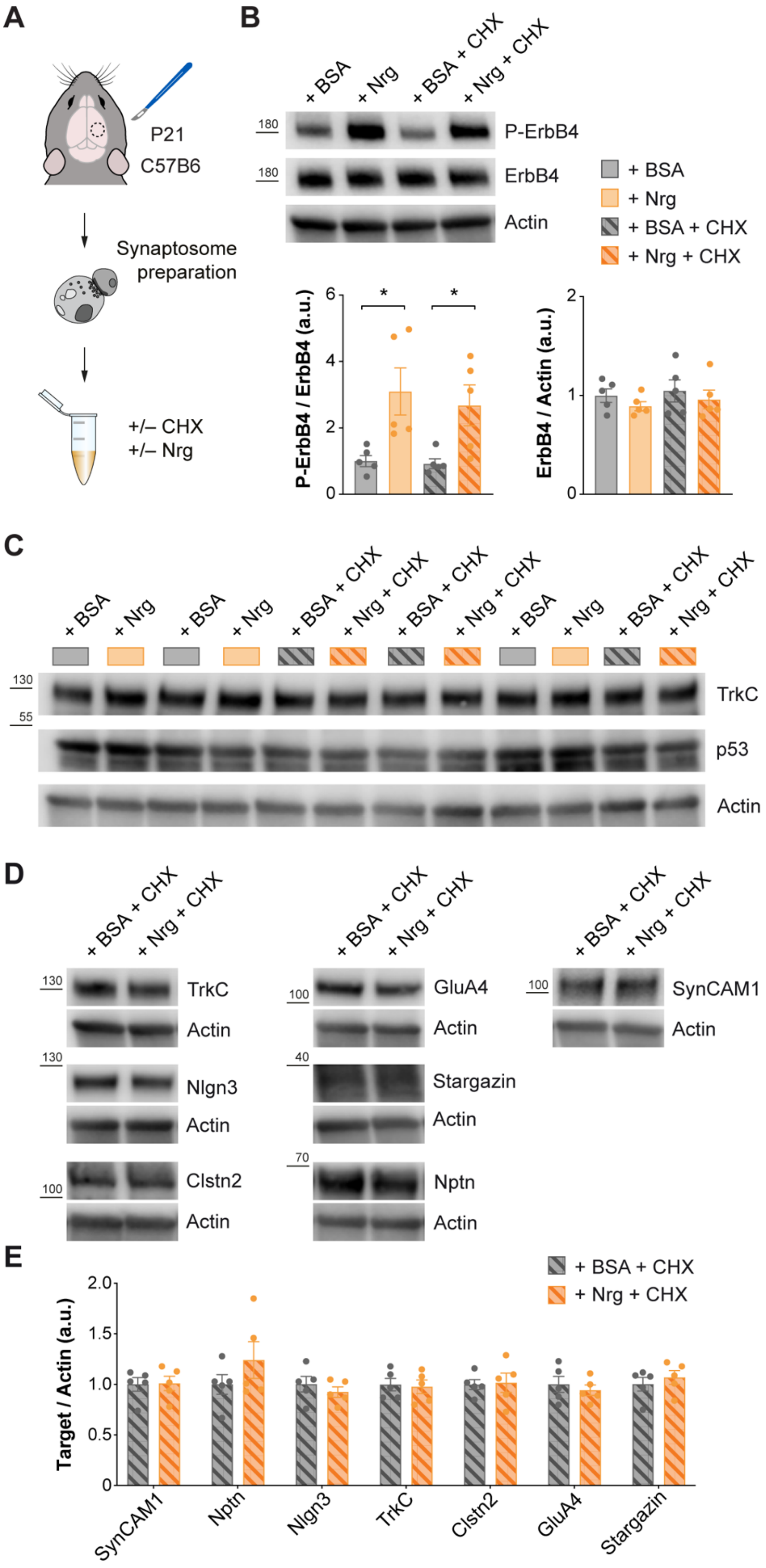
A
P21
C57B6
Synaptosome preparation
+/– CHX
+/– Nrg
B
+ BSA
+ Nrg
+ BSA + CHX
+ Nrg + CHX
P-ErbB4
ErbB4
Actin
P-ErbB4 / ErbB4 (a.u.)
ErbB4 / Actin (a.u.)
C
TrkC
p53
Actin
D
TrkC
Actin
Nlgn3
Clstn2
GluA4
Stargazin
Nptn
SynCAM1
E
Target / Actin (a.u.)
SynCAM1
Nptn
Nlgn3
TrkC
Clstn2
GluA4
Stargazin

Fig. S18. ErbB4 synaptic signaling regulates protein synthesis of synaptic proteins. (**A**) Schematic of experimental design. (**B**) Phosphorylation and protein expression of ErbB4 and actin assessed by Western blot (top) of P21 cortical synaptic fractions treated with Neuregulin (+ Nrg) or control (+ BSA) with or without cycloheximide (+ CHX). Quantification of phosphorylation of ErbB4 normalized to its total expression (bottom left). Quantification of expression levels of ErbB4 normalized to actin (bottom right). One-way ANOVA followed by Tukey's multiple comparisons test or Kruskal-Wallis followed by Dunn's multiple comparisons test: $*P < 0.05$ (all conditions, $n = 5$ synaptosomes). (**C**) Example of a Western blot showing expression of proteins that are changed (TrkC) or not changed (p53) following treatment with Neuregulin (+ Nrg) or control (+ BSA) with or without cycloheximide (+ CHX). (**D**) Protein expression of SynCAM, Nptn, Nlgn3, TrkC, Clstn2, GluA4, Stargazin and actin assessed by Western blot of P21 cortical synaptic fractions treated with Neuregulin (+ Nrg) or control (+ BSA) following pretreatment with cycloheximide (+ CHX). (**E**) Quantification of expression levels of SynCAM, Nptn, Nlgn3, TrkC, Clstn2, GluA4 and Stargazin normalized to actin. Two-tailed Student's unpaired t-tests (all conditions, $n = 5$ synaptosomes). Data are mean ± s.e.m.

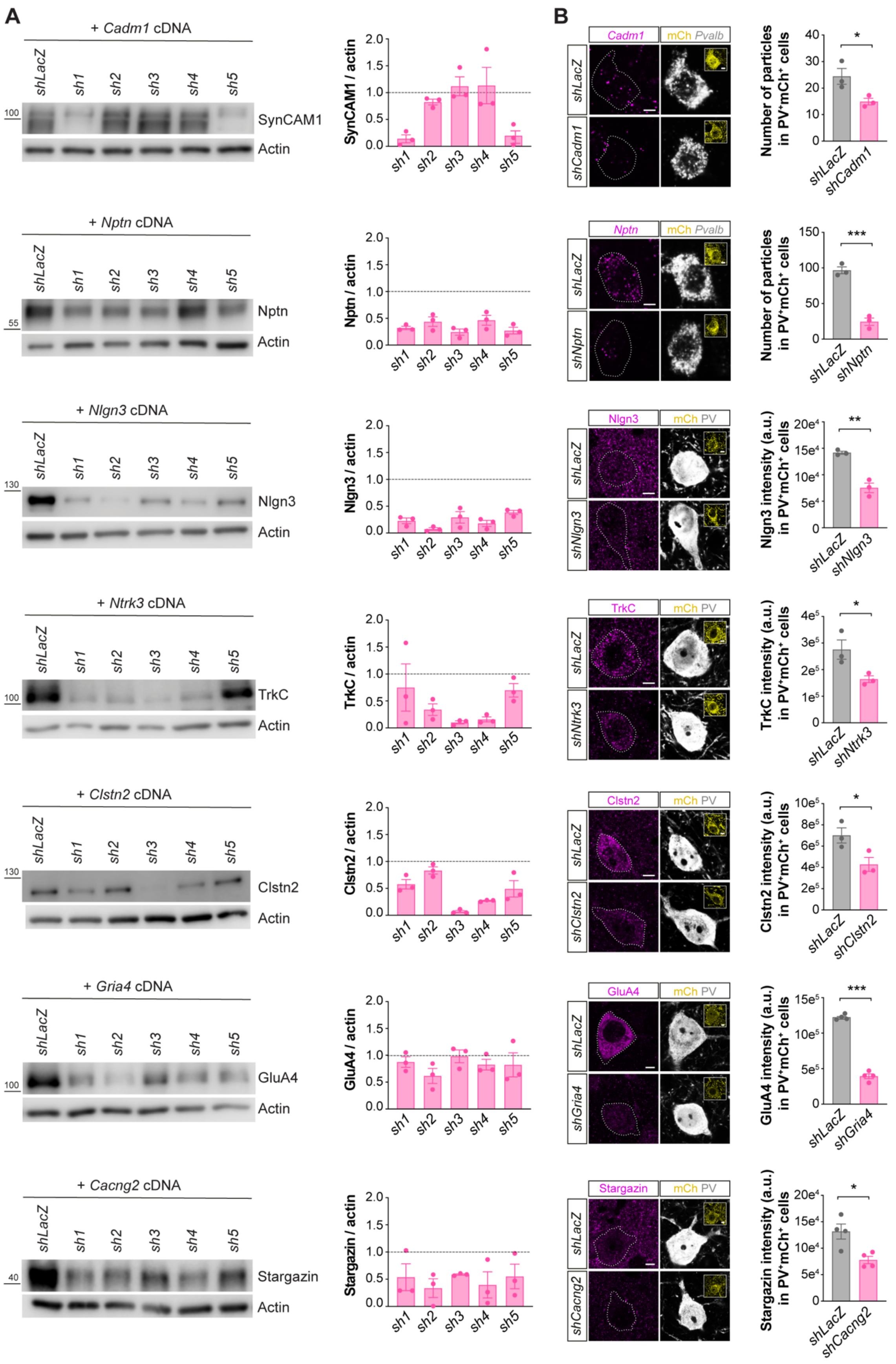
A
B
+ Cadm1 cDNA
shLacZ sh1 sh2 sh3 sh4 sh5
SynCAM1
Actin
SynCAM1 / actin
Cadm1
mCh Pvalb
shCadm1
Number of particles in PV+mCh+ cells
+ Nptn cDNA
Nptn
Nptn / actin
shNptn
+ Nlgn3 cDNA
Nlgn3
Nlgn3 / actin
mCh PV
shNlgn3
Nlgn3 intensity (a.u.) in PV+mCh+ cells
+ Ntrk3 cDNA
TrkC
TrkC / actin
shNtrk3
TrkC intensity (a.u.) in PV+mCh+ cells
+ Clstn2 cDNA
Clstn2
Clstn2 / actin
shClstn2
Clstn2 intensity (a.u.) in PV+mCh+ cells
+ Gria4 cDNA
GluA4
GluA4 / actin
shGria4
GluA4 intensity (a.u.) in PV+mCh+ cells
+ Cacng2 cDNA
Stargazin
Stargazin / actin
shCacng2
Stargazin intensity (a.u.) in PV+mCh+ cells

Fig. S19. In vitro testing and in vivo validation of *shRNA* efficiency for downregulation of target targets. (**A**) In vitro protein expression assessed by Western blot of HA-tagged constructs of the seven target targets from cells transfected with expression plasmids encoding the different targets and plasmids expressing *shRNAs* targeting the corresponding genes (left). Quantification of protein signal normalized to actin for each *shRNA*, relative to control transfections with a plasmid expressing a *LacZ*-targeting *shRNA* (n = 3 wells for each *shRNA*) (right). (**B**) Confocal images illustrating the knockdown of target RNAs (for *Cadm1* and *Nptn*) or target proteins (for Nlgn3, TrkC, Clstn2, GluA4 and Stargazin) in mCh^+ (yellow) PV^+ (grey) cells from P21 *Lhx6-Cre* mice injected with viruses expressing *shRNAs* targeting the genes of interest or with a control virus (*shLacZ*) (left). Quantification of RNA particles (for *Cadm1* and *Nptn*) or target protein staining intensity (for Nlgn3, TrkC, Clstn2, GluA4 and Stargazin) in mCh^+ PV^+ interneurons in knockdown and control mice (right). Two-tailed Student's unpaired t-tests: *P < 0.05, **P < 0.01, ***P < 0.001 (*shCadm1*: n = 38 cells from 3 mice, *shLacZ*: n = 41 cells from 3 mice; *shNptn*: n = 46 cells from 3 mice, *shLacZ*: n = 55 cells from 3 mice; *shNgln3*: n = 59 cells from 3 mice, *shLacZ*: n = 44 cells from 3 mice; *shNtrk3*: n = 52 cells from 3 mice, *shLacZ*: n = 62 cells from 3 mice; *shClstn2*: n = 58 cells from 3 mice, *shLacZ*: n = 70 cells from 3 mice; *shGluA4*: n = 74 cells from 4 mice, *shLacZ*: n = 74 cells from 4 mice; *shCacng2*: n = 102 cells from 4 mice, *shLacZ*: n = 77 cells from 4 mice). Data are mean ± s.e.m. Scale bar, 5 μm.

Table S1. Summary of data and statistical analyses.

Figure 1	Measurement	Values	N	Statistical test	P-value
Figure 1B	Density of VGluT1^{+}PSD95^{+} synapses on PV^{+} cells (synapses/μm, mean ± s.e.m.)	Control: 0.10 ± 0.0045; *Lhx6-Cre;Tsc2*$^{F/+}$: 0.14 ± 0.0074; *Lhx6-Cre;Tsc2*$^{F/F}$: 0.23 ± 0.024.	[brains] Control, *n* = 8; *Lhx6-Cre;Tsc2*$^{F/+}$, *n* = 4; *Lhx6-Cre;Tsc2*$^{F/F}$, *n* = 3.	One-way ANOVA with Tukey's multiple comparisons test	(*) P < 0.05, (***) P < 0.001
Figure 1D	Density of VGluT1^{+}PSD95^{+} synapses on SST^{+} cells (synapses/μm, mean ± s.e.m.)	Control: 0.066 ± 0.0086; *Lhx6-Cre;Tsc2*$^{F/+}$: 0.069 ± 0.0076; *Lhx6-Cre;Tsc2*$^{F/F}$: 0.066 ± 0.0071.	[brains] Control, *n* = 4; *Lhx6-Cre;Tsc2*$^{F/+}$, *n* = 3; *Lhx6-Cre;Tsc2*$^{F/F}$, *n* = 5.	One-way ANOVA with Tukey's multiple comparisons test	(n.s.) P > 0.05
Figure 1G (left)	Frequency of sEPSCs from PV^{+} cells (Hz, mean ± s.e.m.)	Control: 12 ± 0.65; *Lhx6-Cre;Tsc2*$^{F/+}$: 16 ± 1.4; *Lhx6-Cre;Tsc2*$^{F/F}$: 24 ± 1.8.	[cells] Control, *n* = 14 from 5 mice; *Lhx6-Cre;Tsc2*$^{F/+}$, *n* = 23 from 8 mice; *Lhx6-Cre;Tsc2*$^{F/F}$, *n* = 14 from 6 mice.	One-way ANOVA with Tukey's multiple comparisons test	(***) P < 0.001
Figure 1G (right)	Amplitude of sEPSCs from PV^{+} cells (pA, mean ± s.e.m.)	Control: 18 ± 0.76; *Lhx6-Cre;Tsc2*$^{F/+}$: 19 ± 0.53; *Lhx6-Cre;Tsc2*$^{F/F}$: 20 ± 0.68.	[cells] Control, *n* = 14 from 5 mice; *Lhx6-Cre;Tsc2*$^{F/+}$, *n* = 23 from 8 mice; *Lhx6-Cre;Tsc2*$^{F/F}$, *n* = 14 from 6 mice.	Kruskal-Wallis with Dunn's multiple comparisons test	(n.s.) P > 0.05
Figure 1I (left)	Frequency of sEPSCs from SST^{+} cells (Hz, mean ± s.e.m.)	control, 8.7 ± 1.2; *Lhx6-Cre;Tsc2*$^{F/+}$, 9.4 ± 2.6; *Lhx6-Cre;Tsc2*$^{F/F}$, 9.4 ± 1.0.	[cells] control, *n* = 9 from 5 mice; *Lhx6-Cre;Tsc2*$^{F/+}$, *n* = 10 from 9 mice; *Lhx6-Cre;Tsc2*$^{F/F}$, *n* = 26 from 7 mice.	Kruskal-Wallis with Dunn's multiple comparisons test	(n.s.) P > 0.05
Figure 1I (right)	Amplitude of sEPSCs from SST^{+} cells (pA, mean ± s.e.m.)	Control: 21 ± 2.4; *Lhx6-Cre;Tsc2*$^{F/+}$: 18 ± 1.2; *Lhx6-Cre;Tsc2*$^{F/F}$: 19 ± 0.83.	[cells] Control, *n* = 9 from 5 mice; *Lhx6-Cre;Tsc2*$^{F/+}$, *n* = 10 from 9 mice; *Lhx6-Cre;Tsc2*$^{F/F}$, *n* = 26 from 7 mice.	Kruskal-Wallis with Dunn's multiple comparisons test	(n.s.) P > 0.05
**Figure 2**	**Measurement**	**Values**	**N**	**Statistical test**	**P-value**
Figure 2D (top)	Phosphorylation of Tsc2, S6rp and 4EBP1 in synaptosomes (a.u., mean ± s.e.m.)	P-Tsc2/Tsc2: *Erbb4*$^{F/F}$, 1 ± 0.06; *Lhx6-Cre;Erbb4*$^{F/F}$, 0.77 ± 0.08. P-S6rp/S6rp: *Erbb4*$^{F/F}$, 1 ± 0.046; *Lhx6-Cre;Erbb4*$^{F/F}$, 0.72 ± 0.071. P-4EBP1/4EBP1: *Erbb4*$^{F/F}$, 1 ± 0.058; *Lhx6-Cre;Erbb4*$^{F/F}$, 0.77 ± 0.079.	[brains] P-Tsc2/Tsc2: *Erbb4*$^{F/F}$, *n* = 8; *Lhx6-Cre;Erbb4*$^{F/F}$, *n* = 6. P-S6rp/S6rp: *Erbb4*$^{F/F}$, *n* = 5; *Lhx6-Cre;Erbb4*$^{F/F}$, *n* = 4. P-4EBP1/4EBP1: *Erbb4*$^{F/F}$, *n* = 7; *Lhx6-Cre;Erbb4*$^{F/F}$, *n* = 5.	Unpaired *t*-test	(*) P < 0.05
Figure 2D (bottom)	Protein levels of Tsc2, S6rp and 4EBP1 in synaptosomes (a.u., mean ± s.e.m.)	Tsc2/Actin: *Erbb4*$^{F/F}$, 1 ± 0.1; *Lhx6-Cre;Erbb4*$^{F/F}$, 0.89 ± 0.093. S6rp/Actin: *Erbb4*$^{F/F}$, 1 ± 0.25; *Lhx6-Cre;Erbb4*$^{F/F}$, 1.2 ± 0.25. 4EBP1/Actin: *Erbb4*$^{F/F}$, 1 ± 0.14; *Lhx6-Cre;Erbb4*$^{F/F}$, 0.70 ± 0.14.	[brains] Tsc2/Actin: *Erbb4*$^{F/F}$, *n* = 8; *Lhx6-Cre;Erbb4*$^{F/F}$, *n* = 6. S6rp/Actin: *Erbb4*$^{F/F}$, *n* = 5; *Lhx6-Cre;Erbb4*$^{F/F}$, *n* = 4. 4EBP1/Actin: *Erbb4*$^{F/F}$, *n* = 6; *Lhx6-Cre;Erbb4*$^{F/F}$, *n* = 5.	Unpaired *t*-test	(n.s.) P > 0.05

Figure 2F (top)	Phosphorylation of Tsc2, S6rp and 4EBP1 in cytosolic fractions (a.u., mean ± s.e.m.)	P-Tsc2/Tsc2: *Erbb4*$^{F/F}$, 1 ± 0.16; *Lhx6-Cre;Erbb4*$^{F/F}$, 0.83 ± 0.2. P-S6rp/S6rp: *Erbb4*$^{F/F}$, 1 ± 0.057; *Lhx6-Cre;Erbb4*$^{F/F}$, 0.82 ± 0.083. P-4EBP1/4EBP1: *Erbb4*$^{F/F}$, 1 ± 0.15; *Lhx6-Cre;Erbb4*$^{F/F}$, 0.88 ± 0.15.	[brains] P-Tsc2/Tsc2: *Erbb4*$^{F/F}$, *n* = 7; *Lhx6-Cre;Erbb4*$^{F/F}$, *n* = 6. P-S6rp/S6rp: *Erbb4*$^{F/F}$, *n* = 7; *Lhx6-Cre;Erbb4*$^{F/F}$, *n* = 6. P-4EBP1/4EBP1: *Erbb4*$^{F/F}$, *n* = 5; *Lhx6-Cre;Erbb4*$^{F/F}$, *n* = 4.	Unpaired *t*-test	(n.s.) P > 0.05
Figure 2F (bottom)	Protein levels of Tsc2, S6rp and 4EBP1 in cytosolic fractions (a.u., mean ± s.e.m.)	Tsc2/Actin: *Erbb4*$^{F/F}$, 1 ± 0.094; *Lhx6-Cre;Erbb4*$^{F/F}$, 1.1 ± 0.31. S6rp/Actin: *Erbb4*$^{F/F}$, 1 ± 0.079; *Lhx6-Cre;Erbb4*$^{F/F}$, 0.97 ± 0.097. 4EBP1/Actin: *Erbb4*$^{F/F}$, 1 ± 0.26; *Lhx6-Cre;Erbb4*$^{F/F}$, 1.3 ± 0.24.	[brains] Tsc2/Actin: *Erbb4*$^{F/F}$, *n* = 7; *Lhx6-Cre;Erbb4*$^{F/F}$, *n* = 6. S6rp/Actin: *Erbb4*$^{F/F}$, *n* = 7; *Lhx6-Cre;Erbb4*$^{F/F}$, *n* = 6. 4EBP1/Actin: *Erbb4*$^{F/F}$, *n* = 5; *Lhx6-Cre;Erbb4*$^{F/F}$, *n* = 4.	Unpaired *t*-test	(n.s.) P > 0.05
Figure 2H (top)	P-S6rp staining intensity in Nrg3^{+}PSD95^{+} synaptosomes (a.u.)	*Erbb4*$^{F/F}$, 169 ± 13; *Lhx6-Cre;Erbb4*$^{F/F}$, 124 ± 5.6.	[brains] *Erbb4*$^{F/F}$, *n* = 6; *Lhx6-Cre;Erbb4*$^{F/F}$, *n* = 5.	Unpaired *t*-test	(*) P < 0.05
Figure 2H (bottom)	P-S6rp staining intensity in VGluT1^{+}PSD95^{+} synaptosomes (a.u.)	*Erbb4*$^{F/F}$, 146 ± 16; *Lhx6-Cre;Erbb4*$^{F/F}$, 132 ± 16.	[brains] *Erbb4*$^{F/F}$, *n* = 5; *Lhx6-Cre;Erbb4*$^{F/F}$, *n* = 6.	Unpaired *t*-test	(n.s.) P > 0.05
**Figure 4**	**Measurement**	**Values**	**N**	**Statistical test**	**P-value**
Figure 4C	Protein levels of SynCAM1, Nptn, Nlgn3, TrkC, Clstn2, GluA4 and Stargazin in synaptosomes (a.u.)	SynCAM1/Actin: control, 1 ± 0.06; +Nrg, 1.3 ± 0.079. Nptn/Actin: control, 1 ± 0.056; +Nrg, 1.2 ± 0.086. Nlgn3/Actin: control, 1 ± 0.043; +Nrg, 1.1 ± 0.023. TrkC/Actin: control, 1 ± 0.053; +Nrg, 1.2 ± 0.097. Clstn2/Actin: control, 1 ± 0.071; +Nrg, 1.2 ± 0.051. GluA4/Actin: control, 1 ± 0.034; +Nrg, 1.2 ± 0.075. Stargazin/Actin: control, 1 ± 0.043; +Nrg, 1.2 ± 0.064.	[synaptosomes] SynCAM1/Actin: control, *n* = 11; +Nrg, *n* = 11. Nptn/Actin: control, *n* = 9; +Nrg, n=10. Nlgn3/Actin: control, *n* = 4; +Nrg, *n* = 4. TrkC/Actin: control, *n* = 11; +Nrg, *n* = 11. Clstn2/Actin: control, *n* = 4; +Nrg, *n* = 4. GluA4/Actin: control, *n* = 9; +Nrg, n=10. Stargazin/Actin: control, *n* = 7; +Nrg, *n* = 8.	Unpaired *t*-test	(*) P < 0.05, (**) P < 0.01

Figure 5	Measurement	Values	N	Statistical test	P-value
Figure 5C	Density of VGluT1⁺PSD95⁺ synapses on PV⁺ mCh⁺ cells (synapses/µm, mean ± s.e.m.)	SynCAM: *shLacZ*, 0.17 ± 0.011; *shCadm1*, 0.089 ± 0.0067. Nptn: *shLacZ*, 0.16 ± 0.0053; *shNptn*, 0.12 ± 0.0054. Nlgn3: *shLacZ*, 0.17 ± 0.0092; *shNlgn3*, 0.13 ± 0.0054. TrkC: *shLacZ*, 0.17 ± 0.0087; *shNtrk3*, 0.13 ± 0.0077. Clstn2: *shLacZ*, 0.17 ± 0.0067; *shClstn2*, 0.12 ± 0.0089. GluA4: *shLacZ*, 0.18 ± 0.015; *shGria4*, 0.14 ± 0.0055. Stargazin: *shLacZ*, 0.17 ± 0.012; *shCacng2*, 0.14 ± 0.0058.	[brains] SynCAM: *shLacZ*, *n* = 6; *shCadm1*, n=6. Nptn: *shLacZ*, *n* = 6; *shNptn*, n=6. Nlgn3: *shLacZ*, *n* = 4; *shNlgn3*, n=4. TrkC: *shLacZ*, *n* = 5; *shNtrk3*, n=6. Clstn2: *shLacZ*, *n* = 6; *shClstn2*, n=6. GluA4: *shLacZ*, *n* = 5; *shGria4*, n=8. Stargazin: *shLacZ*, *n* = 6; *shCacng2*, n=6.	Unpaired *t*-test	(*) P < 0.05, (**) P < 0.01, (***) P < 0.001
**Figure S1**	**Measurement**	**Values**	**N**	**Statistical test**	**P-value**
Figure S1B (top)	Tsc2 staining intensity in PV⁺ cells (a.u., mean ± s.e.m.)	Control, 587 ± 119; *Lhx6-Cre;Tsc2$^{F/+}$*, 266 ± 44; *Lhx6-Cre;Tsc2$^{F/F}$*, 21 ± 3.4.	[brains] Control, *n* = 4; *Lhx6-Cre;Tsc2$^{F/+}$*, *n* = 4; *Lhx6-Cre;Tsc2$^{F/F}$*, *n* = 4.	One-way ANOVA with Tukey's multiple comparisons test	(*) P < 0.05, (**) P < 0.01
Figure S1B (bottom)	Tsc2 staining intensity in SST⁺ cells (a.u., mean ± s.e.m.)	Control, 404 ± 99; *Lhx6-Cre;Tsc2$^{F/+}$*, 221 ± 37; *Lhx6-Cre;Tsc2$^{F/F}$*, 19 ± 1.9.	[brains] Control, *n* = 4; *Lhx6-Cre;Tsc2$^{F/+}$*, *n* = 4; *Lhx6-Cre;Tsc2$^{F/F}$*, *n* = 4.	One-way ANOVA with Tukey's multiple comparisons test	(**) P < 0.01,
Figure S1D (top)	P-S6rp staining intensity in PV⁺ cells (a.u., mean ± s.e.m.)	Control, 1.0 ± 0.095; *Lhx6-Cre;Tsc2$^{F/+}$*, 1.7 ± 0.35; *Lhx6-Cre;Tsc2$^{F/F}$*, 4.3 ± 0.33.	[brains] Control, *n* = 8; *Lhx6-Cre;Tsc2$^{F/+}$*, *n* = 4; *Lhx6-Cre;Tsc2$^{F/F}$*, *n* = 3.	One-way ANOVA with Tukey's multiple comparisons test	(***) P < 0.001
Figure S1D (bottom)	P-S6rp staining intensity in SST⁺ cells (a.u., mean ± s.e.m.)	Control, 1.0 ± 0.33; *Lhx6-Cre;Tsc2$^{F/+}$*, 1.6 ± 0.54; *Lhx6-Cre;Tsc2$^{F/F}$*, 10 ± 2.9.	[brains] Control, *n* = 3; *Lhx6-Cre;Tsc2$^{F/+}$*, *n* = 3; *Lhx6-Cre;Tsc2$^{F/F}$*, *n* = 3.	One-way ANOVA with Tukey's multiple comparisons test	(*) P < 0.05
Figure S1E (top)	PV⁺ cell volume (µm^3, mean ± s.e.m.)	Control, 864 ± 77; *Lhx6-Cre;Tsc2$^{F/+}$*, 1180 ± 63; *Lhx6-Cre;Tsc2$^{F/F}$*, 1749 ± 52.	[brains] Control, *n* = 8; *Lhx6-Cre;Tsc2$^{F/+}$*, *n* = 4; *Lhx6-Cre;Tsc2$^{F/F}$*, *n* = 3.	One-way ANOVA with Tukey's multiple comparisons test	(*) P < 0.05, (**) P < 0.01, (***) P < 0.001
Figure S1E (bottom)	SST⁺ cell volume (µm^3, mean ± s.e.m.)	Control, 604 ± 66; *Lhx6-Cre;Tsc2$^{F/+}$*, 746 ± 80; *Lhx6-Cre;Tsc2$^{F/F}$*, 1686 ± 172.	[brains] Control, *n* = 3; *Lhx6-Cre;Tsc2$^{F/+}$*, *n* = 3; *Lhx6-Cre;Tsc2$^{F/F}$*, *n* = 3.	One-way ANOVA with Tukey's multiple comparisons test	(**) P < 0.01
**Figure S2**	**Measurement**	**Values**	**N**	**Statistical test**	**P-value**
Figure S2B (top)	Density of PV⁺ cells (cells/mm^2, mean ± s.e.m.)	Control, 225 ± 6.2; *Lhx6-Cre;Tsc2$^{F/+}$*, 223 ± 8.8; *Lhx6-Cre;Tsc2$^{F/F}$*, 215 ± 5.6.	[brains] Control, *n* = 3; *Lhx6-Cre;Tsc2$^{F/+}$*, *n* = 3; *Lhx6-Cre;Tsc2$^{F/F}$*, *n* = 3.	Kruskal-Wallis with Dunn's multiple comparisons test	(n.s.) P > 0.05
Figure S2B (middle)	Density of SST⁺ cells (cells/mm^2, mean ± s.e.m.)	Control, 105 ± 3.2; *Lhx6-Cre;Tsc2$^{F/+}$*, 100 ± 4; *Lhx6-Cre;Tsc2$^{F/F}$*, 101 ± 2.5.	[brains] Control, *n* = 3; *Lhx6-Cre;Tsc2$^{F/+}$*, *n* = 3; *Lhx6-Cre;Tsc2$^{F/F}$*, *n* = 3.	Kruskal-Wallis with Dunn's multiple comparisons test	(n.s.) P > 0.05
Figure S2B (bottom)	Density of PV⁺SST⁺ cells (cells/mm^2, mean ± s.e.m.)	Control, 4.9 ± 0.27; *Lhx6-Cre;Tsc2$^{F/+}$*, 5.5 ± 0.38; *Lhx6-Cre;Tsc2$^{F/F}$*, 13 ± 0.78.	[brains] Control, *n* = 3; *Lhx6-Cre;Tsc2$^{F/+}$*, *n* = 3; *Lhx6-Cre;Tsc2$^{F/F}$*, *n* = 3.	One-way ANOVA with Tukey's multiple comparisons test	(***) P < 0.001

Figure S2C (top)	Laminar distribution of PV^+ cells (cells/mm^2, mean ± s.e.m.)	Layer I: Control, 0 ± 0, *Lhx6-Cre;Tsc2$^{F/+}$*, 0 ± 0; *Lhx6-Cre;Tsc2$^{F/F}$*, 0 ± 0; Layers II/III: Control, 217 ± 15.8, *Lhx6-Cre;Tsc2$^{F/+}$*, 216 ± 9.4; *Lhx6-Cre;Tsc2$^{F/F}$*, 191 ± 9.8; Layer IV: Control, 348 ± 10.0, *Lhx6-Cre;Tsc2$^{F/+}$*, 325 ± 13.4; *Lhx6-Cre;Tsc2$^{F/F}$*, 321 ± 14.8; Layer V: Control, 289 ± 8.7, *Lhx6-Cre;Tsc2$^{F/+}$*, 283 ± 12.7; *Lhx6-Cre;Tsc2$^{F/F}$*, 284 ± 8.0; Layer VI: Control, 148 ± 11.5, *Lhx6-Cre;Tsc2$^{F/+}$*, 158 ± 4.67; *Lhx6-Cre;Tsc2$^{F/F}$*, 156 ± 2.89.	[brains] Control, *n* = 3; *Lhx6-Cre;Tsc2$^{F/+}$*, *n* = 3; *Lhx6-Cre;Tsc2$^{F/F}$*, *n* = 3.	Two-way ANOVA with Tukey's multiple comparisons test	(n.s.) P > 0.05
Figure S2C (middle)	Laminar distribution of SST^+ cells (cells/mm^2, mean ± s.e.m.)	Layer I: Control, 23.0 ± 4.6, *Lhx6-Cre;Tsc2$^{F/+}$*, 22.1 ± 4.7; *Lhx6-Cre;Tsc2$^{F/F}$*, 18.6 ± 1.1; Layers II/III: Control, 86.8 ± 2.6, *Lhx6-Cre;Tsc2$^{F/+}$*, 90.5 ± 6.9; *Lhx6-Cre;Tsc2$^{F/F}$*, 88.8 ± 6.9; Layer IV: Control, 98.4 ± 2.9, *Lhx6-Cre;Tsc2$^{F/+}$*, 96.1 ± 4.2; *Lhx6-Cre;Tsc2$^{F/F}$*, 94.5 ± 2.4; Layer V: Control, 152 ± 4.4, *Lhx6-Cre;Tsc2$^{F/+}$*, 137 ± 6.7; *Lhx6-Cre;Tsc2$^{F/F}$*, 150 ± 2.2; Layer VI: Control, 105 ± 8.5, *Lhx6-Cre;Tsc2$^{F/+}$*, 96.4 ± 4.2; *Lhx6-Cre;Tsc2$^{F/F}$*, 91.3 ± 1.3.	[brains] Control, *n* = 3; *Lhx6-Cre;Tsc2$^{F/+}$*, *n* = 3; *Lhx6-Cre;Tsc2$^{F/F}$*, *n* = 3.	Two-way ANOVA with Tukey's multiple comparisons test	(n.s.) P > 0.05
Figure S2C (bottom)	Laminar distribution of PV^+SST^+ cells (cells/mm^2, mean ± s.e.m.)	Layer I: Control, 0 ± 0, *Lhx6-Cre;Tsc2$^{F/+}$*, 0 ± 0; *Lhx6-Cre;Tsc2$^{F/F}$*, 0 ± 0; Layers II/III: Control, 3.78 ± 0.3, *Lhx6-Cre;Tsc2$^{F/+}$*, 3.66 ± 0.3; *Lhx6-Cre;Tsc2$^{F/F}$*, 5.24 ± 0.4; Layer IV: Control, 9.06 ± 0.9, *Lhx6-Cre;Tsc2$^{F/+}$*, 10.0 ± 0.4; *Lhx6-Cre;Tsc2$^{F/F}$*, 22.8 ± 0.2; Layer V: Control, 5.97 ± 1.2, *Lhx6-Cre;Tsc2$^{F/+}$*, 7.58 ± 1.5; *Lhx6-Cre;Tsc2$^{F/F}$*, 19.4 ± 3.0; Layer VI: Control, 3.11 ± 1.4, *Lhx6-Cre;Tsc2$^{F/+}$*, 3.56 ± 0.5; *Lhx6-Cre;Tsc2$^{F/F}$*, 9.45 ± 1.2.	[brains] Control, *n* = 3; *Lhx6-Cre;Tsc2$^{F/+}$*, *n* = 3; *Lhx6-Cre;Tsc2$^{F/F}$*, *n* = 3.	Two-way ANOVA with Tukey's multiple comparisons test	(**) P , 0.01, (***) P < 0.001

Figure S3	Measurement	Values	N	Statistical test	P-value
Figure S3B	Density of VGluT1⁺PSD95⁺ synapses on PV⁺ dendrites (synapses/µm, mean ± s.e.m.)	Control, 0.2 ± 0.0078; *Lhx6-Cre;Tsc2*$^{F/+}$, 0.2 ± 0.0049; *Lhx6-Cre;Tsc2*$^{F/F}$, 0.25 ± 0.011.	[brains] Control, *n* = 3; *Lhx6-Cre;Tsc2*$^{F/+}$, *n* = 3; *Lhx6-Cre;Tsc2*$^{F/F}$, *n* = 4.	One-way ANOVA with Tukey's multiple comparisons test	(*) P < 0.05
Figure S3C	Density of VGluT1⁺PSD95⁺ synapses on SST⁺ dendrites (synapses/µm, mean ± s.e.m.)	Control, 0.16 ± 0.014; *Lhx6-Cre;Tsc2*$^{F/+}$, 0.16 ± 0.016; *Lhx6-Cre;Tsc2*$^{F/F}$, 0.15 ± 0.009.	[brains] Control, *n* = 3; *Lhx6-Cre;Tsc2*$^{F/+}$, *n* = 3; *Lhx6-Cre;Tsc2*$^{F/F}$, *n* = 4.	One-way ANOVA with Tukey's multiple comparisons test	(n.s.) P > 0.05
**Figure S4**	**Measurement**	**Values**	**N**	**Statistical test**	**P-value**
Figure S4B (top left)	VGluT1⁺ bouton size on PV⁺ cells (µm², mean ± s.e.m.)	Control, 0.5 ± 0.021; *Lhx6-Cre;Tsc2*$^{F/F}$, 0.54 ± 0.016.	[brains] Control, *n* = 4; *Lhx6-Cre;Tsc2*$^{F/F}$, *n* = 5.	Unpaired *t*-test	(n.s.) P > 0.05
Figure S4B (top right)	PSD95⁺ cluster size in PV⁺ cells (µm², mean ± s.e.m.)	Control, 0.23 ± 0.011; *Lhx6-Cre;Tsc2*$^{F/F}$, 0.23 ± 0.011.	[brains] Control, *n* = 4; *Lhx6-Cre;Tsc2*$^{F/F}$, *n* = 5.	Unpaired *t*-test	(n.s.) P > 0.05
Figure S4B (bottom left)	VGluT1⁺ bouton size on SST⁺ cells (µm², mean ± s.e.m.)	Control, 0.39 ± 0.022; *Lhx6-Cre;Tsc2*$^{F/F}$, 0.38 ± 0.013.	[brains] Control, *n* = 4; *Lhx6-Cre;Tsc2*$^{F/F}$, *n* = 5.	Unpaired *t*-test	(n.s.) P > 0.05
Figure S4B (bottom right)	PSD95⁺ cluster size in SST⁺ cells (µm², mean ± s.e.m.)	Control, 0.28 ± 0.0063; *Lhx6-Cre;Tsc2*$^{F/F}$, 0.3 ± 0.011.	[brains] Control, *n* = 4; *Lhx6-Cre;Tsc2*$^{F/F}$, *n* = 5.	Unpaired *t*-test	(n.s.) P > 0.05
**Figure S5**	**Measurement**	**Values**	**N**	**Statistical test**	**P-value**
Figure S5C (left)	Frequency of mEPSCs from PV⁺ cells (Hz, mean ± s.e.m.)	Control: 16.4 ± 1.55; *Lhx6-Cre;Tsc2*$^{F/F}$: 16 ± 1.27.	[cells] Control, *n* = 12 from 7 mice; *Lhx6-Cre;Tsc2*$^{F/F}$, *n* = 10 from 4 mice.	Unpaired *t*-test	(n.s.) P > 0.05
Figure S5C (right)	Amplitude of mEPSCs from PV⁺ cells (pA, mean ± s.e.m.)	Control: 16.8 ± 0.61; *Lhx6-Cre;Tsc2*$^{F/F}$: 17.1 ± 0.73.	[cells] Control, *n* = 12 from 7 mice; *Lhx6-Cre;Tsc2*$^{F/F}$, *n* = 10 from 4 mice.	Unpaired *t*-test	(n.s.) P > 0.05
Figure S5E (left)	Frequency of mEPSCs from SST⁺ cells (Hz, mean ± s.e.m.)	Control: 3.8 ± 1.13; *Lhx6-Cre;Tsc2*$^{F/F}$: 4.3 ± 1.03.	[cells] Control, *n* = 9 from 6 mice; *Lhx6-Cre;Tsc2*$^{F/F}$, *n* = 8 from 4 mice.	Mann-Whitney test	(n.s.) P > 0.05
Figure S5E (right)	Amplitude of mEPSCs from SST⁺ cells (pA, mean ± s.e.m.)	Control: 14 ± 0.78; *Lhx6-Cre;Tsc2*$^{F/F}$: 16 ± 1.10.	[cells] Control, *n* = 9 from 6 mice; *Lhx6-Cre;Tsc2*$^{F/F}$, *n* = 8 from 4 mice.	Unpaired *t*-test	(*) P < 0.05
Figure S5G	PPR from PV⁺ cells (mean ± s.e.m.)	Control: 1.2 ± 0.068; *Lhx6-Cre;Tsc2*$^{F/F}$: 1.3 ± 0.098.	[cells] Control, *n* = 11 from 7 mice; *Lhx6-Cre;Tsc2*$^{F/F}$, *n* = 12 from 6 mice.	Mann-Whitney test	(n.s.) P > 0.05

Figure S7	Measurement	Values	N	Statistical test	P-value
Figure S7B	Density of $Syt2^+Geph^+$ synapses on pyramidal cells (synapses/μm, mean ± s.e.m.)	Control, 0.057 ± 0.0072; *Lhx6-Cre;Tsc2*$^{F/+}$, 0.055 ± 0.011; *Lhx6-Cre;Tsc2*$^{F/F}$, 0.06 ± 0.011.	[brains] Control, *n* = 7; *Lhx6-Cre;Tsc2*$^{F/+}$, *n* = 4; *Lhx6-Cre;Tsc2*$^{F/F}$, *n* = 3.	Kruskal-Wallis with Dunn's multiple comparisons test	(n.s.) P > 0.05
**Figure S9**	**Measurement**	**Values**	**N**	**Statistical test**	**P-value**
Figure S9B	Percentage of particles per field of view (mean ± s.e.m.)	Presynapse, 63.3 ± 1.04; membrane only, 36.7 ± 1.04	[brains] *C57B6*, *n* = 4		
Figure S9D	Percentage of $VGluT1^+PSD95^+$ synaptosomes (mean ± s.e.m.)	Pre with post, 79 ± 0.7; pre only, 21 ± 0.7; post with pre, 64 ± 1.2; post only, 36 ± 1.2	[brains] *C57B6*, *n* = 4		
Figure S9F	Percentage of bipartite synaptosomes (mean ± s.e.m.)	Intact postsynapse, 81 ± 7.7; burst postsynapse, 19 ± 7.7	[micrographs] *C57B6*, *n* = 2 (synaptosome preparations from 4 mice)		
**Figure S12**	**Measurement**	**Values**	**N**	**Statistical test**	**P-value**
Figure S12B	P-S6rp staining intensity in $Syt2^+Geph^+$ synaptosomes (a.u.)	*Erbb4*$^{F/F}$, 130 ± 17; *Lhx6-Cre;Erbb4*$^{F/F}$, 125 ± 23.	[brains] *Erbb4*$^{F/F}$, *n* = 6; *Lhx6-Cre;Erbb4*$^{F/F}$, *n* = 6.	Mann-Whitney test	(n.s.) P > 0.05
**Figure S13**	**Measurement**	**Values**	**N**	**Statistical test**	**P-value**
Figure S13B	Density of $VGluT1^+PSD95^+$ synapses on PV^+ cells (synapses/μm, mean ± s.e.m.)	Control, 0.17 ± 0.012; *Lhx6-Cre;Erbb4*$^{F/F}$, 0.093 ± 0.015; *Lhx6-Cre;Erbb4*$^{F/F}$*;Tsc2*$^{F/+}$, 0.16 ± 0.011	[brains] Control, *n* = 5; *Lhx6-Cre;Erbb4*$^{F/F}$, *n* = 3; *Lhx6-Cre;Erbb4*$^{F/F}$*;Tsc2*$^{F/+}$, *n* = 4.	One-way ANOVA with Tukey's multiple comparisons test	(*) P < 0.05, (**) P < 0.01
**Figure S16**	**Measurement**	**Values**	**N**	**Statistical test**	**P-value**
Figure S16B	Number of particles in PV^+ cells (mean ± s.e.m.)	*Cadm1*, 20 ± 1.3; *Nptn*, 54 ± 2.8; *Nlgn3*, 26 ± 2.1; *Ntrk3*, 26 ± 0.64; *Clstn2*, 31 ± 2.4; *Gria4*, 48 ± 0.68; *Cacng2*, 45 ± 4.	[brains] *C57B6*, *n* = 3		
Figure S16D	Proportion of clusters contacted by $Nrg3^+$ boutons in PV^+ cells (mean ± s.e.m.)	SynCAM1, 84 ± 4.3; Nptn, 71 ± 2.0; Nlgn3, 69 ± 2.9; TrkC, 58 ± 0.82; Clstn2, 61 ± 1.1; GluA4, 45 ± 4,3; Stargazin, 51 ± 2.3.	[brains] *CD1* IUE with HA-Nrg3 plasmid, *n* = 4		
**Figure S18**	**Measurement**	**Values**	**N**	**Statistical test**	**P-value**
Figure S16B (left)	Phosphorylation of ErbB4 in synaptosomes (a.u., mean ± s.e.m.)	P-ErbB4/ErbB4: +BSA, 1 ± 0.168; +Nrg, 3.096 ± 0.710; +BSA+CHX, 0.92 ± 0.147; +Nrg+CHX, 2.68 ± 0.615	[synaptosomes] +BSA, *n* = 5; +Nrg, *n* = 5; +BSA+CHX, *n* = 5; +Nrg+CHX, *n* = 5.	Kruskal-Wallis with Dunn's multiple comparisons test	(*) P < 0.05
Figure S18B (right)	Protein levels of ErbB4 in synaptosomes (a.u., mean ± s.e.m.)	ErbB4/Actin: +BSA, 1 ± 0.068; +Nrg, 0.892 ± 0.045; +BSA+CHX, 1.048 ± 0.112; +Nrg+CHX, 0.96 ± 0.095	[synaptosomes] +BSA, *n* = 5; +Nrg, *n* = 5; +BSA+CHX, *n* = 5; +Nrg+CHX, *n* = 5.	One-way ANOVA with Tukey's multiple comparisons test	(n.s.) P > 0.05

Figure S18E	Protein levels of SynCAM1, Nptn, Nlgn3, TrkC, Clstn2, GluA4 and Stargazin in synaptosomes (a.u.)	SynCAM1/Actin: +BSA+CHX, 1 ± 0.068; +Nrg+CHX, 1.01 ± 0.071. Nptn/Actin: +BSA+CHX, 1 ± 0.097; +Nrg+CHX, 1.24 ± 0.181. Nlgn3/Actin: +BSA+CHX, 1 ± 0.077; +Nrg+CHX, 0.924 ± 0.052. TrkC/Actin: +BSA+CHX, 1 ± 0.062; +Nrg+CHX, 0.976 ± 0.068. Clstn2/Actin: +BSA+CHX, 1 ± 0.049; +Nrg+CHX, 1.02 ± 0.095. GluA4/Actin: +BSA+CHX, 1 ± 0.079; +Nrg+CHX, 0.942 ± 0.053. Stargazin/Actin: +BSA+CHX, 1 ± 0.068; +Nrg+CHX, 1.07 ± 0.068.	[synaptosomes] +BSA+CHX, *n* = 5; +Nrg+CHX, *n* = 5.	Unpaired *t*-test	(n.s.) P > 0.05
**Figure S19**	**Measurement**	**Values**	**N**	**Statistical test**	**P-value**
Figure S19A	Protein levels of HA-SynCAM1, HA-Nptn, HA-Nlgn3, HA-TrkC, HA-Clstn2, HA-GluA4 and HA-Stargazin in HEK293 cells (a.u.)	SynCAM1/Actin: *sh1*, 0.14 ± 0.073; *sh2*, 0.83 ± 0.054; *sh3*, 1.1 ± 0.18; *sh4*, 1.1 ± 0.34; *sh5*, 0.2 ± 0.11. Nptn/Actin: *sh1*, 0.32 ± 0.031; *sh2*, 0.44 ± 0.089; *sh3*, 0.24 ± 0.057; *sh4*, 0.46 ± 0.092; *sh5*, 0.27 ± 0.063. Nlgn3/Actin: *sh1*, 0.23 ± 0.052; *sh2*, 0.075 ± 0. 24; *sh3*, 0.29 ± 0.11; *sh4*, 0.18 ± 0.055; *sh5*, 0.38 ± 0.039. TrkC/Actin: *sh1*, 0.75 ± 0.44; *sh2*, 0.34 ± 0.11; *sh3*, 0.1 ± 0.028; *sh4*, 0.16 ± 0.046; *sh5*, 0.7 ± 0.13. Clstn2/Actin: *sh1*, 0.58 ± 0.088; *sh2*, 0.83 ± 0.067; *sh3*, 0.073 ± 0.021; *sh4*, 0.27 ± 0.01; *sh5*, 0.49 ± 0.15. GluA4/Actin: *sh1*, 0.87 ± 0.1; *sh2*, 0.62 ± 0.14; *sh3*, 0.98 ± 0.12; *sh4*, 0.83 ± 0.098; *sh5*, 0.82 ± 0.22. Stargazin/Actin: *sh1*, 0.54 ± 0.25; *sh2*, 0.34 ± 0.17; *sh3*, 0.59 ± 0.012; *sh4*, 0.4 ± 0.24; *sh5*, 0.55 ± 0.23.	[wells] SynCAM1: for each *shRNA*, *n* = 3; Nptn: for each *shRNA*, *n* = 3; Nlgn3: for each *shRNA*, *n* = 3; TrkC: for each *shRNA*, *n* = 3; Clstn2: for each *shRNA*, *n* = 3; GluA4: for each *shRNA*, *n* = 3; Stargazin: for each *shRNA*, *n* = 3.	One-way ANOVA with Tukey's multiple comparisons test	(*) P < 0.05, (**) P < 0.01, (***) P < 0.001
